# New Labeled PET Analogues Enable the Functional Screening and Characterization of PET-Degrading Enzymes

**DOI:** 10.1021/acssuschemeng.4c00143

**Published:** 2024-04-01

**Authors:** George Taxeidis, Milica Djapovic, Efstratios Nikolaivits, Veselin Maslak, Jasmina Nikodinovic-Runic, Evangelos Topakas

**Affiliations:** † Industrial Biotechnology & Biocatalysis Group, Biotechnology Laboratory, School of Chemical Engineering, National Technical University of Athens, Heroon Polytechniou 9, Zografou, 15772 Athens, Greece; ‡ Faculty of Chemistry, University of Belgrade, Studentski trg 12-16, 11000 Belgrade, Belgrade, Serbia; § Institute of Molecular Genetics and Genetic Engineering, University of Belgrade, Vojvode Stepe 444a, 11000 Belgrade, Serbia

**Keywords:** polyethylene terephthalate, PETase, plastic degradation, screening, fluorescent substrates, kinetics

## Abstract

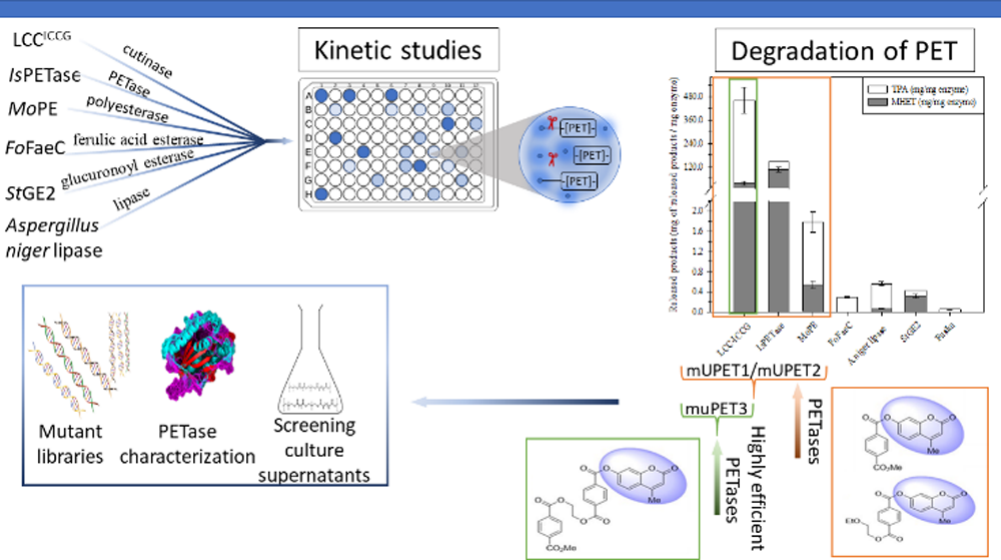

The discovery and engineering of novel biocatalysts capable of depolymerizing polyethylene terephthalate (PET) have gained significant attention since the need for green technologies to combat plastic pollution has become increasingly urgent. This study focuses on the development of novel substrates that can indicate enzymes with PET hydrolytic activity, streamlining the process of enzyme evaluation and selection. Four novel substrates, mimicking the structure of PET, were chemically synthesized and labeled with fluorogenic or chromogenic moieties, enabling the direct analysis of candidate enzymes without complex preparatory or analysis steps. The fluorogenic substrates, mUPET1, mUPET2, and mUPET3, not only identify enzymes capable of PET breakdown but also differentiate those with exceptional performance on the polymer, such as the benchmark PETase, LCC^ICCG^. Among the substrates, the chromogenic *p*-NPhPET3 stands out as a reliable tool for screening both pure and crude enzymes, offering advantages over fluorogenic substrates such as ease of assay using UV–vis spectroscopy and compatibility with crude enzyme samples. However, ferulic acid esterases and mono-(2-hydroxyethyl) terephthalate esterases (MHETases), which exhibit remarkably high affinity for PET oligomers, also show high catalytic activity on these substrates. The substrates introduced in this study hold significant value in the function-based screening and characterization of enzymes that degrade PET, as well as the the potential to be used in screening mutant libraries derived from directed evolution experiments. Following this approach, a rapid and dependable assay method can be carried out using basic laboratory infrastructure, eliminating the necessity for intricate preparatory procedures before analysis.

## Abstract 2

The labeled PET analogues enable rapid function-based screening and characterization of enzymes that degrade PET.

## Introduction

Plastic pollution is a global problem that poses significant harm to the environment due to the nonbiodegradable nature of plastics. These materials can persist in the environment for hundreds of years, leading to the accumulation of plastic waste in landfills, oceans, and other ecosystems.^1,2^ Additionally, they can break down into microplastics, which are prone to ingestion by marine organisms and subsequently enter the food chain, posing potential risks to human health.^1,3,4^ In recent years, there has been growing interest in developing sustainable solutions for managing plastic waste, including enzymatic degradation and recycling. Enzymes can be employed to break down plastic waste into its constituent monomers, facilitating the creation of a circular economy.^5^ This approach offers several advantages over conventional disposal methods, such as incineration or landfill, which introduce secondary pollutants into the environment.

Poly(ethylene terephthalate) (PET) is among the most commonly used plastics, finding applications in various fields, from food packaging to textiles. PET waste is a significant contributor to plastic pollution, emphasizing the importance of effective PET-degrading enzymes in reducing plastic waste.^6^ PETases have demonstrated the ability to degrade PET into its monomers, which can then be repurposed for creating new (bio)materials or added-value products.^7,8^ Despite the potential of enzymatic degradation of plastic waste, the discovery and engineering of efficient PETases and other plastic-degrading enzymes remain significant challenges. A recent opinion paper emphasized the importance of exploring new enzyme classes that are able to degrade PET. To enable the discovery of truly novel enzymes, it is essential to employ model substrates that facilitate their rapid identification and characterization, allowing efficient comparisons of their efficiencies.^9^

PETase activity is most commonly assayed on PET as a substrate in various forms, such as amorphous^10,11^ or semicrystalline^12^ PET films or fibers,^13^ with subsequent quantification of the degradation products using high-performance liquid chromatography (HPLC). Although this method is accurate, it is unable to detect oligomers and has limitations in terms of speed, throughput, and ease of setup for the continuous measurement of enzyme kinetics. To enable faster and higher-throughput analyses, alternative spectrophotometric methods have been developed. For instance, bulk UV absorbance measurement of the reaction supernatant has been proposed as a simpler method, but it comes with some disadvantages: depending on the mode of action of the enzyme (if it produces monomers or oligomers), the measurements can be inaccurate and difficult to compare.^14^ Alternatively, the utilization of ketoreductases (KREDs) for the detection of released ethylene glycol (EG) showed a good correlation to the HPLC results.^15^ Another approach for quantifying PET degradation products, initially introduced by Ebersbach et al., converts products into fluorescent compounds through a mechanism driven by the iron autoxidation-mediated generation of free hydroxyl radicals.^16^ However, Zurier and Goddard highlighted that this particular protocol encounters limitations that arise from the fact that the mono-(2-hydroxyethyl) terephthalate (MHET) exhibits a lower fluorescent extinction coefficient compared to that of terephthalic acid (TPA),^13^ so in a hydrolysis mixture where both products are present, TPA will contribute mostly to fluorescence. This discrepancy likely explains why Ebersbach et al. reported HPLC and fluorescent results solely in terms of TPA quantity which was measured after PET nanoparticles' hydrolysis by *Tf*Cut2 from*Thermobifida fusca*KW3.^16^ Taking into account the fact that *Tf*Cut2 yields mostly MHET when acting upon PET nanoparticles (MHET accounts for 75% of the total products),^17^ questions are being raised about the sensitivity of this technique for screening PETases, which predominantly release MHET. This concern has also been addressed by Weigert et al., who improved their assay by adding an MHETase to the mixture to further convert released MHET into TPA. This additional step has the potential to amplify fluorescence intensities; however, it is worth noting that it introduces some complexity into the screening protocol, which may impact its straightforwardness and simplicity.^18^

Despite the aforementioned issues involving product quantification, synthesis of PET nanoparticles demands highly corrosive solvents such as hexafluoro-2-propanol (HFIP),^19^ not to mention that there is currently limited information available regarding their application in metagenome libraries. Other substrates such as PET microfibers^13^ and semicrystalline PET coating^18^ have been also used for high-throughput applications; however, it should be considered that screening protocols using those substrates demand long incubation times (reaching up to 72 h)^13^ or high temperatures (more than 60 °C).^19^ At the same time, model substrates closely resembling the PET structure have also been synthesized throughout the years, with the first being bis(benzoyloxyethyl) terephthalate (3PET). The activity of enzymes on this substrate was correlated to their activity on PET fabrics.^20^ Similarly, bis(*p*-methylbenzoate) (2PET) has been utilized for the development of a turbidimetric assay for high-throughput screening of PET oligomer-hydrolyzing enzymes.^21^ Additionally, the synthesis of a set of PET-related substrates, including the PET dimer, has shown potential for the detection of PETases.^22^ However, these model substrates are mostly insoluble in water, and calculation of kinetic constants on these requires labor-intensive procedures and compromises the application and interpretation of data from simple assays.^14^

Given the proven usefulness of stable chromogenic and fluorogenic compounds for straightforward detection of hydrolytic activities of enzymes and characterization of biocatalysts, this study aimed to synthesize new labeled compounds that can be used as substrates for identifying and characterizing PET-degrading enzymes. These compounds have been designed to mimic the structure of PET and contain chromo- or fluorogenic moieties that enable real-time monitoring of enzyme activity. We evaluated the performance of the fluorogenic substrates by conducting kinetic studies with enzymes known for their activity on PET, including the engineered leaf-branch compost cutinase LCC (ICCG mutant), *Is*PETase from*Ideonella sakaiensis*, and the polyesterase *Mo*PE from *Moraxella* sp., as well as three enzymes that cannot degrade PET as negative controls. The results demonstrated that the fluorescent substrate, mUPET2, can be a valuable tool for functional screening since it can differentiate enzymes with activity on PET, while the bulkiest synthesized substrates (mUPET3) can identify PET-degrading enzymes with high performance on the polymer. Additionally, the study explored the limitations associated with using labeled substrates to assess the activity of enzymes that degrade PET oligomers. Finally, a chromogenic substrate was synthesized and utilized as a reliable tool for screening both pure and crude enzymes. This assay method requires only a simple analytical infrastructure to evaluate the PET-degrading activity.

## Materials and Methods

### Substrates and Chemicals

4-Methylumbelliferone (4-mU) was purchased from Acros Organics (Geel, Belgium), while 4-nitrophenol (*p*-NPhOH) was purchased from Fluka (Buchs, Switzerland). Impranil DLN-SD was obtained from Covestro Solution Center (Leverkusen, Germany). Amorphous PET (*x*_c_ 5%) was cryomilled in a PULVERISETTE 14 (FRITSCH Corp., Idar-Oberstein, Germany) according to the procedure mentioned before,^23^ resulting in particle size <500 μm. 4-Nitrophenyl octanoate (*p*NPh-octanoate) and 4-methylumbelliferone octanoate (4-mU-octanoate) were purchased from Sigma-Aldrich (St Louis, USA) and Santa-Cruz Biotechnology (Santa Cruz, USA), respectively. Dicyclohexylcarbodiimide (DCC) was purchased from Sigma Aldrich; dichloromethane, ethyl acetate, and petroleum ether (60–80 °C) were purchased from Fischer Chemicals (Zurich, Switzerland); pyridine was purchased from Carlo Erba (Milano, Italy). Methyl 4-(chloroformyl)benzoate was purchased from TCI Chemicals (Portland, USA). PET-labeled substrates were synthesized as described below.

### Synthesis of the PET Model Substrates and Structural Analysis

**Figure 1:**
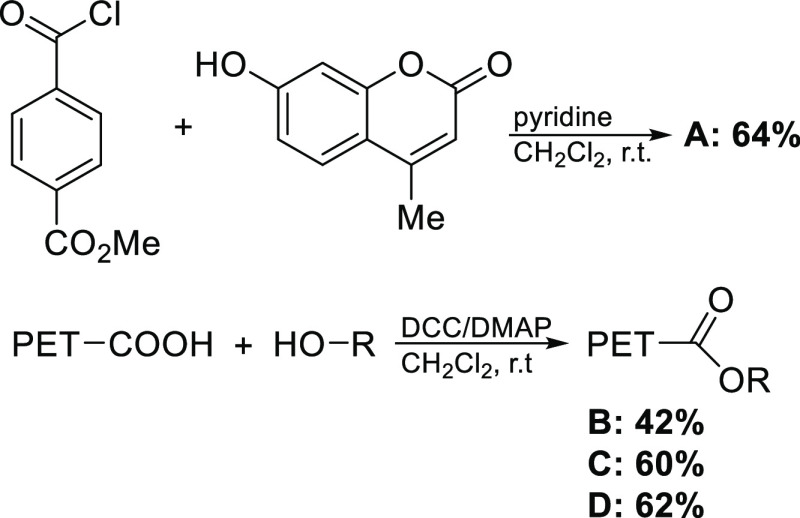
Chemical synthesis of PET model substrates. mUPET1 compound (A) was prepared from methyl 4-(chloroformyl)benzoate and 4-mU, while synthesis of mUPET2 (B), mUPET3 (C), and *p*-NPhPET3 (D) model compounds was performed from PET oligomers and 4-mU or *p*-NPhOH. The conversion yields for A, B, C, and D were 64, 42, 60, and 62%, respectively.

Model compounds with fluorogenic (compounds **A**–**C**) and chromophoric (compound **D**) moieties were synthesized by esterification of PET oligomers. These compounds, synthesized for the first time, were produced using two approaches (Figures 1 and S1): (i) esterification of acids with alcohols in the presence of DCC and (ii) Schotten–Baumann reaction for acylation of alcohols with acyl halide in the presence of organic bases.

All chromatographic separations were performed on Silica 10–18, 60 Å (ICN Biomedicals). Standard techniques were used for the purification of the reagents. ^1^H and ^13^C NMR spectra were recorded with Varian/Agilent NMR 400 MHz (^1^H at 400 MHz, ^13^C at 101 MHz). Chemical shifts (δ) are expressed in ppm, and coupling constant (*J*) in Hz. TMS was used as an internal standard. The following abbreviation was used for signal multiplicities (s = singlet, t = triplet, q = quartet, dd = doublet of doublets, m = multiplet). IR spectra (ATR) were recorded with a PerkinElmer-FT-IR 1725X spectrophotometer; ν values are given in cm^–1^. Mass spectra were obtained on MS LTQ Orbitrap XL. Melting points were determined on the Electrothermal WRS1B apparatus and were reported uncorrected. As the alcohol counterpart in these reactions, we used commercially available compounds *p*-NPhOH and 4-mU. Compound **A** was prepared from methyl 4-(chloroformyl)benzoate and 4-mU (Figure 1). Synthesis of model compounds **B**, **C,** and **D** was performed from PET oligomers that contained a free carboxylic group. These carboxylic acids were prepared according to optimized synthetic procedures (see the Supporting Information). Products **A–D** were isolated as pure compounds and were characterized using NMR (Figures S2–S8), IR spectroscopy, as well as mass spectrometry.

### Preparation of Methyl (4-Methyl-2-oxo-2*H*-chromen-7-yl) Terephthalate **A** (**mUPET1**)

A solution of methyl 4-(chloroformyl)benzoate (224.4 mg; 1.13 mmol; 1 equiv) in dichloromethane (3.5 mL) was added dropwise to a cold (0 °C) suspension of 7-hydroxy-4-methylcoumarin (200.1 mg; 1.13 mmol; 1 equiv) and pyridine (0.9 mL; 1.13 mmol; 1 equiv) in dichloromethane (2.5 mL) over 5 min. The reaction was carried out at room temperature for 16 h. The mixture was diluted with dichloromethane (6.0 mL) and successively washed with water, aqueous solution of Na_2_CO_3_ (5%, w/v), 1 M HCl, and brine. The extract was dried over anhydrous Na_2_SO_4_ and concentrated under vacuum. The crude product was purified by dry flash chromatography (SiO_2_; eluent: dichloromethane/petroleum ether/ethyl acetate = 60:40:3) to afford 244 mg (**64%**) of **mUPET1** as a white solid (mp 195–196 °C).

^1^H NMR (400 MHz, CDCl_3_): δ_H_ 8.27 (d, *J* = 8.3 Hz, 2H), 8.19 (d, *J* = 8.3 Hz, 2H), 7.67 (d, *J* = 8.6 Hz, 1H), 7.26 (s, 1H), 7.22 (dd, *J* = 8.6, 2.1 Hz, 1H), 6.30 (s, 1H), 3.98 (s, 3H), 2.46 (s, 3H). ^13^C NMR (101 MHz, CDCl_3_): δ_C_ 166.2, 163.9, 160.5, 154.4, 153.2, 152.0, 135.0, 132.7, 130.4, 129.9, 125.7, 118.3, 118.2, 114.9, 110.7, 52.7, 18.9. IR (ATR) *v*_max_: 3064, 2956, 1725,1615,1572, 1501, 1436, 1408, 1389, 1371, 1262, 1153, 1108, 1085, 1107, 981, 874, 720. HRMS: *m*/*z* [M + Na]^+^ calcd for C_19_H_14_O_6_: 361.0683; found: 361.0680.

### Preparation of 2-Ethoxyethyl (4-Methyl-2-oxo-2*H*-chromen-7-yl) Terephthalate **B** (**mUPET2**), 2-((4-(Methoxycarbonyl)benzoyl)oxy)ethyl (4-Methyl-2-oxo-2*H*-chromen-7-yl) Terephthalate **C** (**mUPET3**), and 2-((4-(Methoxycarbonyl)benzoyl)oxy)ethyl (4-Nitrophenyl) Terephthalate **D** (**p-NPhPET3**)

A solution of DCC (1.14 mmol; 1.2 equiv) in dichloromethane (3 mL) was added dropwise to a suspension of PET-COOH (0.94 mmol; 1 equiv), 4-mU or p-NPhOH (1.40 mmol; 1.5 equiv), 4-dimethyl-aminopyridine (DMAP) (0.47 mmol; 0.5 equiv), and dichloromethane (11 mL). The reaction mixture was stirred overnight at room temperature. Dicyclohexylurea was separated by filtration, and the filtrate was washed with an aqueous solution of Na_2_CO_3_ (5%, w/v) (2 × 5 mL), 1 M HCl (1 × 5 mL), and saturated aqueous NaCl solution. The organic layer was dried over anhydrous Na_2_SO_4_, and the solvent was removed by using a vacuum evaporator. The crude product was purified by dry flash chromatography, and products A, B, and C were obtained in the form of a white solid.

Product **B** (**mUPET2**) was obtained as a white solid (mp = 114–116 °C) according to the aforementioned procedure (156 mg, **42%**).

^1^H NMR (400 MHz, CDCl_3_): δ_H_ 8.27 (d, *J* = 8.2 Hz, 2H), 8.21 (d, *J* = 8.4 Hz, 2H), 7.67 (d, *J* = 8.6 Hz, 1H), 7.27 (s, 1H), 7.22 (dd, *J* = 8.6, 2.3 Hz, 1H), 6.30 (s, 1H), 4.54–4.50 (m, 2H), 3.82–3.78 (m, 2H), 3.60 (q, *J* = 7.0 Hz, 2H), 2.46 (s, 3H), 1.24 (t, *J* = 7.0 Hz, 3H). ^13^C NMR (101 MHz, CDCl_3_): δ_C_ 165.7, 163.9, 160.5, 154.4, 153.2, 152.0, 135.1, 132.7, 130.4, 130.1, 125.7, 118.3, 118.2, 114.9, 110.7, 68.4, 66.9, 65.0, 18.9, 15.3. IR (ATR) *v*_max_: 3056, 2986, 2900, 2879, 1720, 1627, 1612, 1503, 1423, 1389, 1287, 1251, 1123, 1112, 1084, 1052, 881, 721. HRMS: *m*/*z* [M + Na]^+^ calcd for C_22_H_20_O_7_: 419.1101; found: 419.1101

Product **C** (**mUPET3**) was obtained as a white solid (mp = 168–170 °C) according to the aforementioned procedure (299 mg, **60%**).

^1^H NMR (400 MHz, CDCl_3_): 8.27 (d, *J* = 8.5 Hz, 2H), 8.19 (d, *J* = 8.5 Hz, 2H), 8.14–8.08 (m, 4H), 7.67 (d, *J* = 8.6 Hz, 1H), 7.25 (d, *J* = 2.3 Hz, 1H), 7.21 (dd, *J* = 8.6, 2.2 Hz, 1H), 6.30 (d, *J* = 0.8 Hz, 1H), 4.73 (s, 4H), 3.94 (s, 3H), 2.46 (d, *J* = 0.9 Hz, 3H). ^13^C NMR (101 MHz, CDCl_3_): δ_C_ 166.3, 165.7, 165.4, 163.8, 160.5, 154.4, 153.2, 152.0, 134.6, 134.3, 133.6, 132.9, 130.5, 130.1, 129.8, 129.7, 125.7, 118.3, 118.2, 114.9, 110.7, 63.3, 63.1, 52.6, 18.9. IR (ATR) *v*_max_: 2956, 2924, 1746, 1722, 1627, 1610, 1573, 1500, 1442, 1408, 1385, 1368, 1337, 1248, 1147, 1118, 1079, 977, 874, 721. HRMS: *m*/*z* [M + Na]^+^ calcd for C_29_H_22_O_10_: 553.1105; found: 553.1107

Product **D** (*p***-NPhPET3**) was obtained as a white solid (mp = 158–160 °C) according to the aforementioned procedure (287.3 mg, **62%**).

^1^H NMR (400 MHz, CDCl_3_): δ_H_ 8.37–8.31 (m, 2H), 8.27 (d, *J* = 8.5 Hz, 2H), 8.20 (d, *J* = 8.5 Hz, 2H), 8.14–8.09 (m, 4H), 7.46–7.41 (m, 2H), 4.73 (s, 4H), 3.95 (s, 3H). ^13^C NMR (101 MHz, CDCl_3_): δ_C_ 166.3, 165.7, 165.4, 163.5, 155.5, 145.8, 134.8, 134.4, 133.6, 132.7, 130.5, 130.1, 129.8, 129.8, 125.5, 122.7, 63.4, 63.1, 52.6. IR (ATR) *v*_max_: 3085, 2963, 1745, 1720, 1614, 1592, 1578, 1519, 1491, 1435, 1411, 1344, 1279, 1255, 1210, 1118, 1076, 1022, 883, 722. HRMS: *m*/*z* [M + Na]^+^ calcd for C_25_H_19_NO_10_: 516.0906; found: 516.0914

### Preparation of Recombinant Enzymes and Native Enzyme Mixtures

The enzymes used in this study were the leaf-branch compost cutinase ICCG variant (LCC^ICCG^),^24^*Is*PETase from *I. sakaiensis*,^25^ the polyesterase from *Moraxella* sp. (*Mo*PE),^23^ the tannase-like ferulic acid esterase (FAE) from*Fusarium oxysporum*(*Fo*FaeC),^26^ the glucuronoyl esterase from*Thermothelomyces thermophila*(*St*GE2),^27^ and a commercial lipase from*Aspergillus niger*(CAS No.: 9001-62-1, EC 3.1.1.3, Fluka Analytical, Switzerland).

*Is*PETase, LCC^ICCG^, and *Mo*PE were expressed in*Escherichia coli*BL21 cells harboring a recombinant pET-22b(+) vector (Novagen, St. Louis, USA) and purified from the intracellular fraction, as previously described.^28^*Fo*FaeC and *St*GE2 were expressed in*Komagataella phaffii*(*Pichia pastoris*) and purified as described before.^26,27^

The purity of the isolated enzymes was confirmed by 12% sodium dodecyl sulfate-polyacrylamide gel electrophoresis (SDS-PAGE), and protein concentration was determined spectrophotometrically (*A*_280nm_) based on each enzyme’s molar extinction coefficient (*Is*PETase: 39,670 M^–1^ cm^–1^, LCC^ICCG^: 28,836 M^–1^ cm^–1^, *Mo*PE: 47,245 M^–1^ cm^–1^, *Fo*FaeC: 112,020 M^–1^ cm^–1^, *St*GE2: 46,002 M^–1^ cm^–1^) calculated by the ProtParam tool from ExPASy.^29^ Fractions containing the purified enzymes were dialyzed overnight at 4 °C against a 20 mM Tris–HCl buffer (pH 7.5).

The culture supernatant from*F. oxysporum*BPOP18 (*Fus*Im), which shows polyesterase-degrading activity, was also tested as a candidate for PETase activity. The extracellular enzyme preparation was induced by Impranil DLN-SD (0.4% v/v), which was used as a sole carbon source in the culture medium, as described elsewhere.^30^ Protein concentration in the culture supernatant was estimated according to the Lowry assay,^31^ while quantification was performed using bovine serum albumin solution as a standard.

### Enzymatic Hydrolysis of Virgin PET and Detection of Degradation Products

Purified enzymes’ ability to break down PET was determined after mixing 10 mg of amorphous PET powder with 50 μg of each enzyme in 1 mL of 0.1 M phosphate buffer pH 7, while in the case of *Fus*Im, the protein amount used for PET hydrolysis was 7 μg. The reactions were kept under agitation (1200 rpm) in an Eppendorf Thermomixer Comfort (Eppendorf, Germany) at 30 °C for 3 days, and every 24 h, half of the initial amount of each enzyme was added to the reaction mixture. The degradation products were detected and quantified in the Agilent 1260 Infinity II instrument (Agilent Technologies, Germany), using an Agilent 1260 Infinity II variable wavelength detector (VWD) (G7114A), following the protocol described elsewhere.^23^

### Determination of Enzyme Kinetic Constants Using Fluorogenic PET Model Substrates

**Figure 2:**
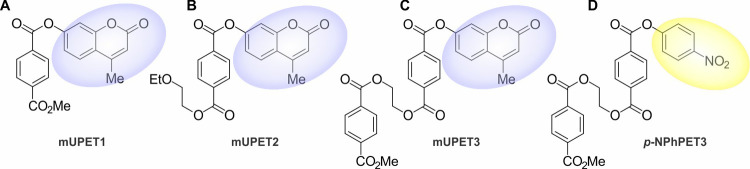
Structure of model substrates mUPET1 (A), mUPET2 (B), mUPET3 (C), and *p*-NPhPET3 (D). The chromogenic and fluorogenic moieties, which are released due to enzymatic hydrolysis, are depicted in yellow and blue circles, respectively. The release of 4-mU or *p*-NPhOH is monitored through fluorescence (A, B, C) and UV–vis spectroscopy (D).

Stock solutions of fluorogenic PET model substrates (mUPET1, mUPET2, and mUPET3; Figure 2) were prepared in DMSO at a concentration of 5 mM. Reactions were conducted in a total volume of 100 μL, using 0.1 M phosphate buffer at pH 7. Each substrate was mixed with 10 μL of the respective enzyme, and the necessary amount of DMSO was added to maintain a final concentration of 5% (v/v). The concentrations of mUPET1, mUPET2, and mUPET3 ranged from 0 to 30, 50, and 70 μM, respectively, while the enzyme quantity was varied to obtain a linear reaction curve for the course of the assay. Control reactions were performed after replacing the volume of the enzyme solution with an equal amount of 20 mM Tris–HCl (pH 7.5).

Reactions were performed at 30 °C for 15 min in a Tecan Infinite M1000 Pro fluorescence microplate reader (Switzerland), equipped with the analysis software Tecan i-control 1.11. Fluorescence was recorded every 30 s, setting excitation and emission wavelengths at 380 and 454 nm,^32^ respectively. Fluorescence was converted to 4-mU concentration via a standard curve constructed with pure 4-mU in 0.1 M phosphate buffer, pH 7. One unit of enzyme activity is defined as the amount of enzyme releasing 1 μmol of 4-mU per min.

The kinetic constants presented in this study were calculated on GraphPad Prism 8 software (GraphPad Software, Boston, USA). The experimental values are presented as means ± standard deviation (SD) of three replicates (*n* = 3). Statistical tests analyzing the difference between the means of each independent kinetic constant were also performed (one-way ANOVA).

### Activity Assay with a Chromogenic PET Model Substrate

Similar to fluorogenic substrates, a 10 mM stock solution of the chromogenic substrate (*p*-NPhPET3) (Figure 2D) was prepared in DMSO. Reactions were initiated after mixing a *p*-NPhPET3 stock solution with 20 μL of each enzyme in a total volume of 250 μL of 0.1 M phosphate buffer pH 7 resulting in a dispersion (59 μg of total substrate amount). The reaction mixture was incubated at 30 °C under agitation (1300 rpm) in an Eppendorf Thermomixer Comfort (Eppendorf, Germany) for 15 min. After 15 min, the reaction tubes were put on ice to minimize enzyme activity and centrifuged at 4000 *g* for 30 s. Enzyme activity was determined by measuring the release of *p*-NPhOH in the reaction supernatant at 410 nm in a SpectaMax ABS Plus microplate reader (Molecular Devices, LLC). A standard curve of *p*-NPhOH in 0.1 M citrate-phosphate buffer at pH 7 was used to quantify the release of *p*-NPhOH in the enzyme reactions. One unit of enzyme activity is defined as the amount of enzyme releasing 1 μmol of *p*-NPhOH per min.

## Results and Discussion

### Selection of Candidate Enzymes and Their Ability To Degrade Amorphous PET

The PET-degrading enzymes used in this study have been previously investigated for PET degradation, demonstrating varying performance. This observation facilitates the assessment of labeled substrates for sorting enzyme candidates based on their PET degradation ability. More specifically, PET hydrolases LCC^ICCG^, *Is*PETase, and *Mo*Pe exhibit different performances in PET depolymerization, allowing their correlation with the labeled substrates. Additionally, *Fo*FaeC is a representative tannase-like FAE^33−35^ that can release hydroxycinnamic acids from hemicellulose and pectin, hydrolyzing the ester bond with l-arabinofuranose-containing polysaccharides.^36^*Is*MHETase from *I. sakaiensis* is the only known MHET esterase, yielding TPA and ethylene glycol.^37^ Despite the low sequence identity shared between *Fo*FaeC and *Is*MHETase (27% for 82% coverage), *Is*MHETase also belongs to FAE-like enzymes^38^ and is a structural homologue of *Fo*FaeC with a *Z*-score of 35.0 and a root-mean-square deviation of 2.6 Å.^34^*Fo*FaeC was selected as an intriguing candidate not only because it could be possibly assayed when conducting functional screening tests for PETases but also due to this enzyme’s capacity to hydrolyze PET oligomers yielding TPA.^23^ Apart from *Fo*FaeC, enzymes hydrolyzing natural polymers were also selected in this study, considering the structural and chemical similarities shared between natural and synthetic polymers.^39^ Glucuronoyl esterases are carbohydrate esterases that have been reported to hydrolyze lignin–carbohydrate ester bonds in lignin–carbohydrate complexes.^40^ To the best of our knowledge, these enzymes have never been tested in synthetic polymer degradation. On the contrary, lipases have long been reported to hydrolyze petroleum-based polyesters,^41^ exhibiting degrading activity on poly(ε-caprolactone) (PCL),^41,42^ polyethylene succinate (PES),^42^ and PET fabrics and films.^43^ Especially for the *A. niger* lipase used in this study, according to Ion et al., this enzyme acts as a BHETase, hydrolyzing BHET to MHET and TPA, with the conversion of MHET to TPA being a time- and enzyme concentration-dependent process.^44^ In pursuit of evaluating a crude enzyme preparation against labeled substrates, the culture supernatant from*F. oxysporum*BPOP18 (*Fus*Im) induced by Impranil DLN-SD was also employed.

**Figure 3:**
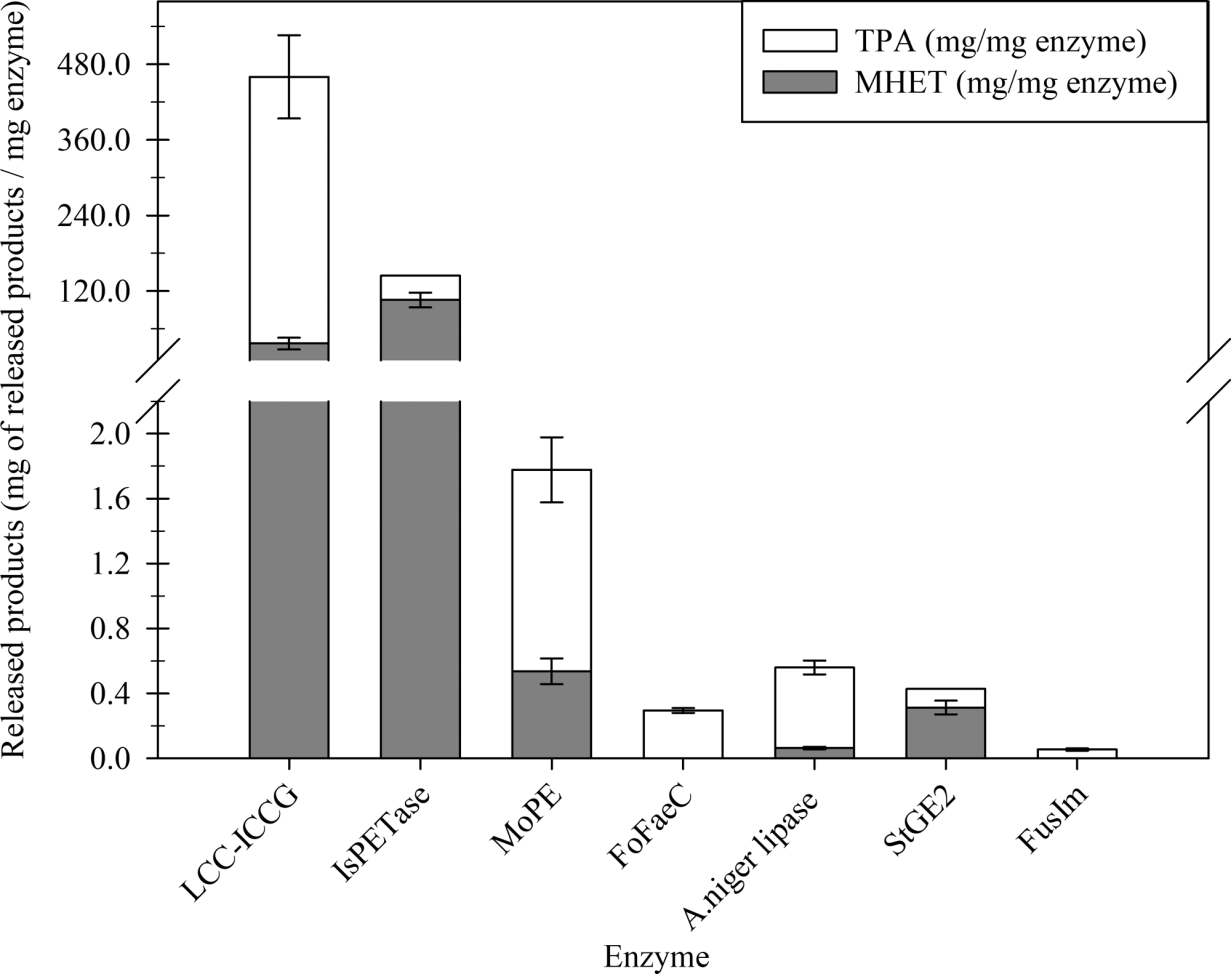
Amount (mg/mg enzyme) of MHET (gray bars) and TPA (white bars) released after treating amorphous PET powder with pure enzymes and a crude enzyme mixture. The PET oligomers were detected though HPLC after a 3-day treatment of PET at 30 °C. No degradation products were detected in control reactions (absence of enzymes).

All of the above-mentioned enzymes were tested for their degrading activity on amorphous PET, and the amount of TPA and MHET released is depicted in Figure 3. Treatment of PET powder with equivalent amounts of known polyesterases led to considerable amounts of monomer release approaching 450 mg/mg of enzyme for LCC^ICCG^, while the corresponding amount in the case of *Mo*PE was almost 300-fold lower. Conversely, *Fo*FaeC,*A. niger* lipase, *St*GE2, and *Fus*Im demonstrated negligible PET degradation activity, releasing no more than 0.54 mg of degradation products per mg of enzyme (Figure 3). These findings will serve as a benchmark for evaluating the efficacy of the labeled substrates in distinguishing PET hydrolases from general ester-cleaving enzymes while classifying PET hydrolases based on their performance on the polymer.

### Fluorogenic Model Substrates' Potential for Assaying PET-Degrading Activity

**Figure 4:**
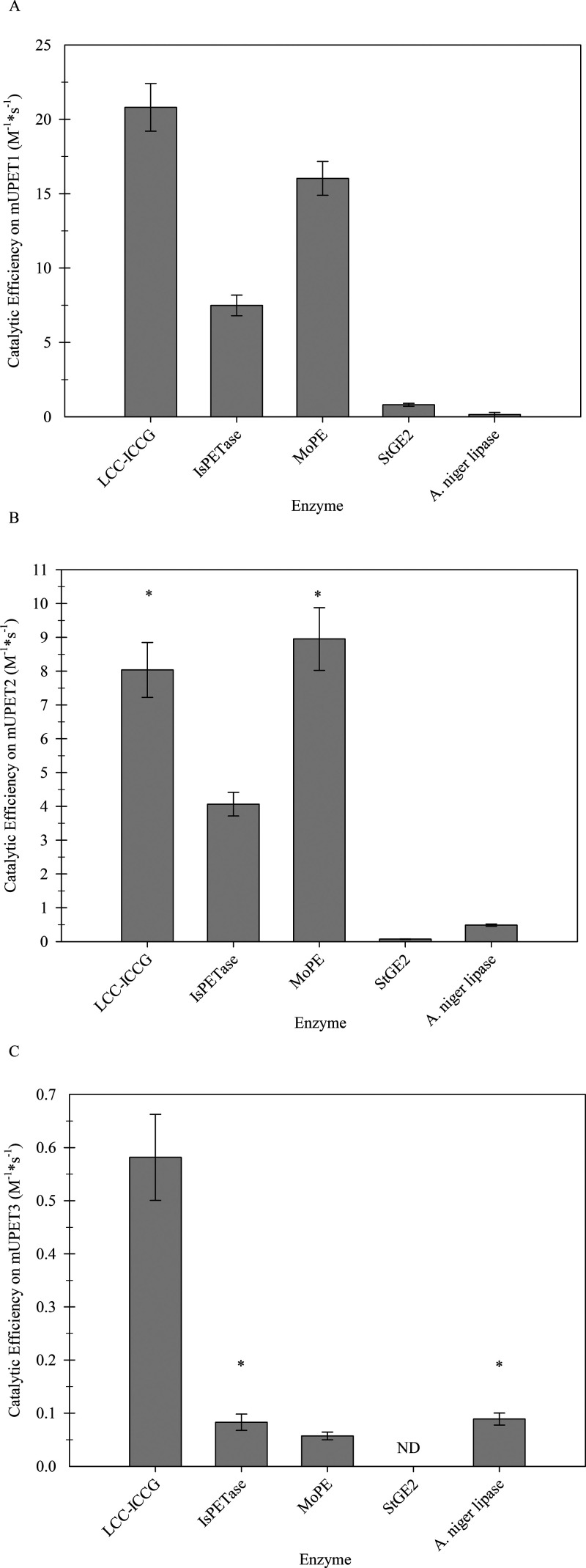
Catalytic efficiency (*k*_cat_/*K*_M_) of each enzyme on mUPET1 (A), mUPET2 (B), and mUPET3 (C). The star symbol (*) indicates no statistical difference between the values.

The kinetic characteristics of the enzymes mentioned above were determined on the fluorescent substrates, where the results revealed variations in the catalytic efficiency (*k*_cat_/*K*_M_) among the different PET hydrolases. For example, in mUPET1 and mUPET2, the catalytic efficiency ranged from 7.5 to 20.8 M^–1^ s^–1^ and 4.1 to 9.0 M^–1^ s^–1^, respectively (Figure 4). In contrast, the other esterases exhibited significantly lower catalytic efficiencies, as the highest *k*_cat_/*K*_M_ value was 9-fold lower in mUPET1 and 25-fold lower in mUPET2 compared to the least efficient PET hydrolase (Figure 4A,B).

When mUPET3 was used as a substrate, the catalytic efficiencies determined ranged from 0.06 to 0.6 M^–1^ s^–1^, which were up to 280-fold lower compared to the other fluorescent substrates (Figure 4C). All enzymes displayed similar *k*_cat_/*K*_M_ values, except for LCC^ICCG^ that exhibited the highest catalytic efficiency, which was up to 10-fold greater than those of the other enzymes. This finding suggests that mUPET3 might be able to differentiate highly efficient PET hydrolases, whereas enzymes with little or no activity on PET, like *St*GE2, have negligible activity on this substrate, which does not allow the calculation of kinetic constants.

In the context of this study, the turnover number (*k*_cat_) was also calculated (Table 1). The *k*_cat_ of *Is*PETase on mUPET1 was found to be 1.5- and 1.7-fold higher compared to those of LCC^ICCG^ and *Mo*PE, respectively, reaching almost 0.5 × 10^–4^ s^–1^. However, a different trend was observed for mUPET2, since the *k*_cat_ of *Mo*PE was up to 2.4-fold higher than those of LCC^ICCG^ and *Is*PETase, whose turnover numbers remained relatively consistent, as those before. In general, it is important to note that the *k*_cat_ values of enzymes with PET-degrading activity are at least 34- and 76-fold higher in mUPET1 and mUPET2, respectively, compared to those of the best-performing esterase.

**Table 1:** *K*_M_ Constant (μM) and *k*_cat_ (s^–1^) of Each Enzyme on mUPET1 (A), mUPET2 (B), and mUPET3 (C)^a^

	*K*_M_ (μM)	*k*_cat_ (s^–1^)
(A) mUPET1		
LCC^ICCG^	1.6 ± 0.1	3.4 × 10^–5^ ± 0.1 × 10^–5^
*Is*PETase	6.7 ± 0.6	5.0 × 10^–5^ ± 0.2 × 10^–5^
*Mo*PE	1.9 ± 0.1	3.0 × 10^–5^ ± 0.1 × 10^–5^
*St*GE2	0.7 ± 0.1	5.7 × 10^–7^ ± 0.1 × 10^–7^
*A. niger* lipase	5.7 ± 0.5	8.7 × 10^–7^ ± 0.1 × 10^–7^
(B) mUPET2		
LCC^ICCG^	7.2 ± 0.7	5.8 × 10^–5^ ± 0.2 × 10^–5^
*Is*PETase	9.3 ± 0.8	3.8 × 10^–5^ ± 0.1 × 10^–5^
*Mo*PE	15.6 ± 1.5	1.4 × 10^–4^ ± 0.1 × 10^–5^
*St*GE2	4.7 ± 0.4	3.4 × 10^–7^ ± 0.1 × 10^–7^
*A. niger* lipase	1.0 ± 0.1	5.0 × 10^–7^ ± 0.1 × 10^–7^
(C) mUPET3		
LCC^ICCG^	21.7 ± 2.8	1.3 × 10^–5^ ± 0.1 × 10^–5^
*Is*PETase	34.1 ± 5.5	2.8 × 10^–6^ ± 0.3 × 10^–6^
*Mo*PE	17.4 ± 2.1	9.9 × 10^–7^ ± 0.5 × 10^–7^
*St*GE2	ND	ND
*A. niger* lipase	0.6 ± 0.1	5.6 × 10^–8^ ± 0.2 × 10^–8^
*A. niger* lipase	0.6 ± 0.1	5.6 × 10^–8^ ± 0.2 × 10^–8^

a ND: not determined.

Furthermore, when examining mUPET3, LCC^ICCG^ exhibited the maximum *k*_cat_, which was 4.5- and 13-fold higher than those of *Is*PETase and *Mo*PE, respectively (Table 1). This reaffirms earlier observations regarding the *k*_cat_/*K*_M_ values (Figure 4), highlighting the superior catalytic efficiency of LCC^ICCG^ for PET degradation. It is noteworthy that the determined turnover numbers for this substrate were at least 10-fold lower than those of mUPET1 and mUPET2. The reduced turnover rates can be attributed to the fact that *k*_cat_ defines the release rate of the fluorescent moiety, and since mUPET3 contains four ester bonds, the cleavage of that specific bond has a slower rate.

Regarding the *K*_M_ values, these can be generally described by the following correlation, *K*_M(mUPET3)_ > *K*_M(mUPET2)_ > *K*_M(mUPET1)_ (Table 1). This pattern could be the result of the elevated hydrophobicity when the substrate size increases and/or the fact that bulkier substrates exhibit greater steric hindrance leading to reduced enzyme affinity. Based on this observation, the *K*_M_ values of LCC^ICCG^ were significantly impacted in comparison to their corresponding values in mUPET1, since its *K*_M_ was increased by 4.5- and 13.4-fold for the hydrolysis of mUPET2 and mUPET3 substrates, respectively.

Regarding *St*GE2 and*A. niger* lipase, the affinity of the glucuronoyl esterase was among the highest in mUPET1 and mUPET2, whereas the affinity of*A. niger* lipase was the greatest in mUPET2 and mUPET3 (Table 1). Although these enzymes exhibit significantly high affinity for these substrates, probably due to esterases’ preference for PET oligomers, neither their catalytic efficiency nor PET powder degradation establishes them as potential PET degraders. Besides, it is known that these enzymes can hydrolyze the ester bond within the PET oligomers without necessarily having the capability to extensively degrade the PET polymer itself.^45,46^ Due to these reasons, *K*_M_ cannot be considered an indicator that can be correlated with enzyme performance on PET polymer, so an enzyme selection should not rely only on this kinetic parameter.

Considering all of the aforementioned kinetic parameters, each substrate can give important information, allowing screening and sorting of the studied enzymes based on their efficiency. Especially for mUPET1 and mUPET2, these substrates could identify the enzymes with activity on PET (Figure 3), as their *k*_cat_ and *k*_cat_/*K*_M_ values were up to 400- and 137-fold higher than those of the other enzymes tested. Regarding mUPET3, the corresponding values proved that this substrate could identify and differentiate highly efficient PET hydrolases such as LCC^ICCG^, not to mention that the esterases with imperceptible activity on PET polymer had either low or no activity on this substrate; a piece of useful evidence as interference of insufficient hydrolases can be avoided.

### Limitations Using Labeled Substrates for Screening PET Hydrolases

The newly designed and synthesized labeled substrates have been demonstrated as novel tools for assaying the PET-degrading activity of characterized enzymes, as they can rapidly identify PET-degrading candidates and even differentiate those with high activity on PET. Aiming to understand any potential limitations that the labeled substrates would pose, the class of PET oligomer-degrading enzymes was investigated. This investigation was motivated by a previous study wherein a novel enzyme called PET46 was discovered through a sequence-based metagenome search for PETases. PET46 shares structural similarities with feruloyl esterases and exhibits the ability to hydrolyze MHET, BHET, and synthetic PET oligomers, including 3PET. However, its activity on PET polymer was significantly lower when compared to benchmark enzymes such as *Is*PETase and LCC.^47^ In this context, we also assessed the ability of the labeled substrates to differentiate between FAEs/MHETases and PETases. Similar observations were also observed in our case, as *Fo*FaeC showed 874-fold, 5-fold, and 9-fold greater catalytic efficiency on mUPET1, mUPET2, and mUPET3, respectively, when compared to the next best-performing enzyme (Tables 1 and 2). The results obtained in our study demonstrate that FAEs can also generate false-positive indications, a fact that should be considered when using mUPET substrates. This limitation may also apply to MHETases considering the high similarity in mode of action to FAEs.

**Table 2:** *K*_M_ Constant (μM), *k*_cat_ (s^–1^), and *k*_cat_/*K*_M_ (M^–1^ s^–1^) of *Fo*FaeC on mUPET Substrates, 4-mU-Octanoate, and *p*-NPh-Octanoate

substrate	*K*_M_ (μM)	*k*_cat_ (s^–1^)	*k*_cat_/*K*_M_ (M^–1^ s^–1^)
mUPET1	1.7 × 10^–3^ ± 0.1 × 10^–3^^a^	30.0 × 10^–3^ ± 0.4 × 10^–3^	18.0 × 10^3^ ± 1.2 × 10^3^
mUPET2	7.5 × 10^–3^ ± 0.6 × 10^–3^	3.8 × 10^–4^ ± 0.1 × 10^–4^	51.0 ± 4.6
mUPET3	2.2 × 10^–3^ ± 0.3 × 10^–3^^a^	11.0 × 10^–6^ ± 0.4 × 10^–6^	5.3 ± 0.7
4-mU-octanoate	1.9 × 10^–3^ ± 0.3 × 10^–3^^a^	2.1 × 10^–3^ ± 0.1 × 10^–3^	12.0 × 10^2^ ± 1.8 × 10^2^
*p*-NPh-octanoate	7.7 × 10^–1^ ± 0.7 × 10^–1^	8.5 × 10^–6^ ± 0.2 × 10^–6^	1.1 × 10^–2^ ± 0.1 × 10^–2^

a The values exhibit no statistically significant difference.

The remarkably high catalytic efficiency of *Fo*FaeC compared to those of the other enzymes tested, alongside the similar *K*_M_ values found in mUPET substrates despite their structural diversity, implies a plausible mechanism whereby *Fo*FaeC preferentially recognizes and cleaves the fluorogenic moiety of the substrate. To further examine this hypothesis, *Fo*FaeC was also assayed with 4-mU- and *p*-NPhOH-octanoate, which is a substrate specified for measuring lipase activity. Interestingly, the catalytic efficiency determined using 4-mU-octanoate as a substrate was more than 100,000-fold higher compared to that obtained using *p*-NPh-octanoate, although the enzyme should in theory recognize the acyl moiety of each substrate. Furthermore, the *K*_M_ values of mUPET1, mUPET3, and 4-mU-octanoate did not exhibit statistically significant differences, indicating similar substrate affinities. Similarly, the *K*_M_ of mUPET2 fell within a similar range, but on the other hand, the *K*_M_ value of *p*-NPh-octanoate was significantly higher. All of these findings collectively indicate that this specific enzyme effectively cleaves the 4-mU moiety, highlighting the limitations of assaying FAEs/MHETases, with 4-mU-labeled substrates. Simultaneously, the much lower affinity and catalytic efficiency of *Fo*FaeC toward the *p*-NPhOH moiety is an observation that will be explored in greater depth within this study, since the utilization of substrates with alternative labeling may potentially enable the differentiation between FAEs/MHETases and actual PETases.

Taking all of the results and limitations into account, it is noteworthy that fluorescent substrates serve as a valuable tool for the rapid identification and assessment of enzymes with PETase activity. Particularly, these labeled substrates are well-suited for screening mutant libraries or characterizing engineered PETases, providing valuable insights into enzyme performance as they can promptly evaluate their activity on PET. Moreover, these substrates enable determination of kinetics without the requirement for additional secondary analyses, thereby making the screening process more efficient. However, it should be mentioned that the same compounds are not recommended when screening a metagenomic library since the possible presence of FAEs/MHETases can result in false-positive indications, necessitating subsequent tests using PET polymer.

In addition to quantifying the enzymatic activities of various 4-mU PET analogues, a quick qualitative assay can be conducted using a 96-well microplate under room temperature conditions and UV light exposure (Figure S9). However, attempts to develop an agar-plate-based screening assay utilizing the 4-mU PET analogues for direct assessment of an *E. coli* mutant library were unsuccessful. One possible explanation for this outcome is the inability of the labeled substrates to penetrate the intracellular space and undergo hydrolysis by the recombinantly expressed enzymes. Hence, it is advisable to utilize an enzyme-secreting expression host for the establishment of an agar plate assay screening.

### Use of a Chromogenic Substrate for PETase Activity Screening

As shown by the kinetic results, mUPET3 proved superior in the identification of enzymes with high PET-degrading activity compared with the other fluorogenic substrates. Additionally, the reduced binding affinity of FAE toward the *p*-NPhOH moiety (Table 2) prompted us to synthesize a new substrate (*p*-NPhPET3), akin to mUPET3, wherein 4-mU was replaced with *p*-NPhOH as a chromophore (Figure 1D). The hydrolysis of *p*-NPhPET3 can be easily assayed by vis spectroscopy, a fact that makes its use more laboratory-friendly, not to mention that it is partially insoluble under assay conditions, resembling the hydrophobic and insoluble structure of PET.

At this point, another notable benefit of UV/vis spectroscopy is its capability to assay a broader range of enzyme preparations, including the microbe’s exoproteome. This exoproteome (or secretome) is another source for discovering novel PETases, although screening with fluorescent substrates is often restrictive according to the principles of fluorescence microscopy. More specifically, screening crude enzymes derived from culture supernatants can interfere with a fluorescent assay, since compounds with significant biological activity can be fluorescent themselves or act as a quencher.^48^ For example, secondary metabolites, such as flavonoids, contribute the most to background fluorescence interfering with the fluorescence emission of 4-mU.^49^ In the case of screening whole cells, it should be considered that NADPH fluorescence wavelength bands (excitation range 320–380 nm and emission range 420–480 nm) overlap with those of 4-mU.^48,50^

**Figure 5:**
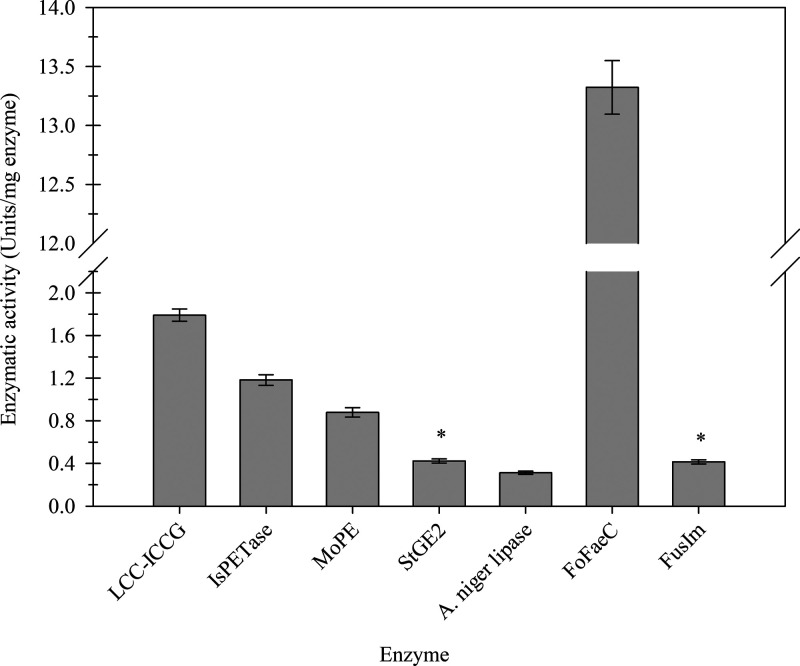
Specific activity (units/mg enzyme) of various enzymes on *p*-NPhPET3. The star symbol (*) indicates no statistical difference between values.

For all the aforementioned reasons, the screening assay utilizing *p*-NPhPET3 included not only purified enzymes but also *Fus*Im, the induced secretome of *F. oxysporum*, wherein various enzymes including lipases, cutinases, and carboxypeptidases have been identified.^30^ These enzymes have putative PETase activity^5^; therefore, *Fus*Im was also included in our study and tested with *p*-NPhPET3. As shown in Figure 5, *Fo*FaeC could extensively hydrolyze *p*-NPhPET3 exceeding 13 units/mg enzyme, verifying again the conclusion that FAEs present particularly high activity on these substrates. Regarding the other enzymes, the activity of LCC^ICCG^, *Is*PETase, and *Mo*PE was approximately 2- to 4-fold higher than that of *St*GE2, which exhibited the highest activity among *A. niger* lipase and *Fu*sIm, reaching 0.45 units/mg enzyme. Furthermore, the activity of *Fu*sIm showed no statistical difference compared to *St*GE2, while the activity of *A. niger* lipase fluctuated at the same level, indicating that the *p*-NPhPET3 substrate could successfully differentiate PET hydrolases from the ester-cleaving enzymes. However, it is worth noting that *Fus*Im is a crude enzyme preparation, leading to a lower ratio of enzymes with actual PETase activity, a fact which potentially underestimates its ability in PET hydrolysis.

Despite the need for a centrifugation step prior to analysis, *p*-NPhPET3 stands out as a useful tool for effectively sorting enzymes based on their PET-degrading ability and allows the screening of both pure and crude enzymes obtained from culture supernatants. Considering the aforementioned limitations, both chromogenic and fluorogenic substrates can be used not only for determining the activity of putative PETases but also as screening tools in high-throughput assays for molecular evolution studies. Also in this case, we observed that the performance of PET hydrolases mirrors that of real PET, with LCC^ICCG^ emerging as the most efficient candidate for PET hydrolysis when assessed with the chromogenic substrate. Regarding other ester-cleaving enzymes, both glucuronoyl esterase *St*Ge2 and*A. niger* lipase demonstrate comparable levels of PET degradation performance and exhibit similar activity in *p*-NPhPET3, whereas *Fus*Im displays a minimal impact on PET degradation despite its activity being comparable to that of *St*Ge2. This discrepancy may be attributed to the presence of other enzymes within the induced secretome, particularly feruloyl esterases, as previously discussed in a study by Taxeidis et al.^30^

Overall, these substrates offer significant advantages in terms of time efficiency and cost-effectiveness as they can reliably and directly determine enzymes with PETase activity. Particularly during enzyme screening, which involves numerous candidates, the substrates can be inexpensively synthesized, significantly reducing both the cost and time required compared to HPLC analysis. Notably, this assay enables kinetic measurements of up to 96 samples, offering a substantial advantage over HPLC, which at the same time can only measure a single time point of one sample.

Moreover, this screening assay demands smaller enzyme amounts compared to PET degradation studies due to the high sensitivity of fluorescence, also providing initial insights into enzyme performance and subsequently reducing reaction times for PET degradation. Finally, considering the high costs associated with purchasing and maintaining the HPLC infrastructure, the economic feasibility of substrate synthesis becomes even more evident, as this method can eliminate the large number of samples needed to be measured with liquid chromatography, further highlighting the advantages of the assay.

## Conclusions

The discovery of new biocatalysts capable of hydrolyzing PET is currently receiving significant attention, as combating plastic pollution using environmentally friendly technologies has emerged as a pressing task for the scientific community. The labeled substrates exemplified in this study serve as valuable indicators for enzymes with PET hydrolytic activity, allowing the identification of PETases with exceptional performance in PET degradation. Particularly, *p*-NPhPET3 stands out as a reliable tool for screening both pure and crude enzymes, offering a straightforward assay protocol that can be performed using a simple analytical infrastructure. However, it is important to exercise caution when using these labeled substrates to assay FAEs/MHETases, as their strong affinity for the labeled moiety and PET oligomers can lead to extensive hydrolysis of the PET-labeled substrates. In summary, the substrates presented in this study are valuable for function-based screening and characterization of PET-degrading enzymes, as well as for high-throughput screening of mutant libraries in directed evolution experiments. This fast and reliable assay methodology simplifies the process by using a simple laboratory infrastructure without the need for complex preparatory steps prior to analysis.

## Supporting Information Available

Kinetic constant values (XLSX)
Experimental procedures for labeled substrates; characterization analyses; and ^1^H NMR spectra (PDF)
