# Correction to “New Labeled PET Analogues Enable the Functional Screening and Characterization of PET-Degrading Enzymes”

**DOI:** 10.1021/acssuschemeng.4c05228

**Published:** 2024-07-03

**Authors:** George Taxeidis, Milica Djapovic, Efstratios Nikolaivits, Veselin Maslak, Jasmina Nikodinovic-Runic, Evangelos Topakas

In our original article, in the “Funding” paragraph, the following APC Funding Statement was added:

The open access publishing of this article is financially supported by HEAL-Link.

