# Supplementary material for: New Labeled PET Analogues Enable the Functional Screening and Characterization of PET-Degrading Enzymes
Source: ACS Sustain Chem Eng. 2024 Apr 1;12(15):5943–52. doi: 10.1021/acssuschemeng.4c00143 (PMC11187625; doi:10.1021/acssuschemeng.4c00143)
Supplement: Supplementary file 2 — sc4c00143_si_002.pdf [file sc4c00143_si_002.pdf]

Author Information

Corresponding Authors

Evangelos Topakas - Industrial Biotechnology & Biocatalysis Group, Biotechnology Laboratory, School of Chemical Engineering, National Technical University of Athens, 9 Heroon Polytechniou str, 15772 Zografou, Athens, Greece

https://orcid.org/0000-0003-0078-5904

Veselin Maslak - University of Belgrade, Faculty of Chemistry, Studentski trg 12-16, 11000 Belgrade, Belgrade, Serbia

https://orcid.org/0000-0002-5735-3953

Authors

George Taxeidis - Industrial Biotechnology & Biocatalysis Group, Biotechnology Laboratory, School of Chemical Engineering, National Technical University of Athens, 9 Heroon Polytechniou str, 15772 Zografou, Athens, Greece

https://orcid.org/0000-0002-2881-7608

Milica Djapovic - University of Belgrade, Faculty of Chemistry, Studentski trg 12-16, 11000 Belgrade, Belgrade, Serbia
https://orcid.org/0000-0002-8264-5739

Efstratios Nikolaivits - Industrial Biotechnology & Biocatalysis Group, Biotechnology Laboratory, School of Chemical Engineering, National Technical University of Athens, 9 Heroon Polytechniou str, 15772 Zografou, Athens, Greece

https://orcid.org/0000-0002-8022-9272

Jasmina Nikodinovic-Runic - Institute of Molecular Genetics and Genetic Engineering, University of Belgrade, Vojvode Stepe 444a, 11000 Belgrade, Serbia
https://orcid.org/0000-0002-2553-977X

Manuscript Title

New labelled PET analogues enable the functional screening and characterization of PET-degrading enzymes

Number of Pages: 13
Number of Figures: 9
Number of Tables: 0

New labelled PET analogues enable the functional screening and characterization of PET-degrading enzymes

George Taxeidis[a], Milica Djapovic[b], Efstratios Nikolaivits[a], Veselin Maslak[b]*, Jasmina Nikodinovic-Runic[c], Evangelos Topakas[a]*

[a]Industrial Biotechnology & Biocatalysis Group, Biotechnology Laboratory, School of Chemical Engineering, National Technical University of Athens, Heroon Polytechniou 9, 15772 Zografou, Athens, Greece

[b]University of Belgrade, Faculty of Chemistry, 1 Studentski trg, 11000 Belgrade, Belgrade, Serbia

[c]Institute of Molecular Genetics and Genetic Engineering, University of Belgrade, Vojvode Stepe 444a, 11000 Belgrade, Serbia

Correspondence to: E. Topakas; vtopakas@chemeng.ntua.gr and V. Maslak; vmaslak@chem.bg.ac.rs

Table of contents

Figure S1. Methodological approach in synthesis of **PET[B]-COOH and PET[C/D]-COOH** precursors……………………………………………………………………………..…S4

Experimental procedures:

4-((2-ethoxyethoxy)carbonyl)benzoic acid (PET[B]-COOH)…………………………… S4

5-((2-((4-(methoxycarbonyl)benzoyl)oxy)ethoxy)carbonyl)benzoic acid (PET[C/D]-COOH) …………………………………………………………………………………S4

Figure S2. ^{1}H-NMR and ^{13}C-NMR spectra of **PET[B]-COOH**…. ………………………….S6

Figure S3. ^{1}H-NMR and ^{13}C-NMR spectra of **PET[C/D]-COOBn**………………………..S7

Figure S4. ^{1}H-NMR and ^{13}C-NMR spectra of **PET[C/D]-COOH**…………………………..S8

Figure S5. ^{1}H-NMR and ^{13}C-NMR spectra of **mUPET1 (A)**………………………………..S9

Figure S6. ^{1}H-NMR and ^{13}C-NMR spectra of **mUPET2 (B)**………………………………S10

Figure S7. ^{1}H-NMR and ^{13}C-NMR spectra of **mUPET3 (C)**………………………………S11

Figure S8. ^{1}H-NMR and ^{13}C-NMR spectra of ***p*-NPhPET3 (D)**……………………………S12

Figure S9. Qualitative assay at room temperature under UV light exposure using 100 µM of mUPET2 as a substrate………………………………………………………………S13

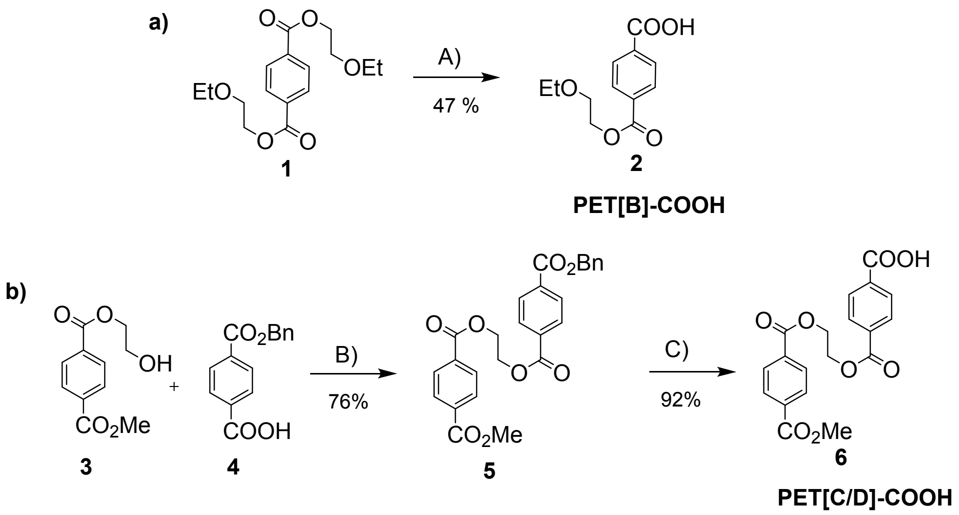

Figure S1. Methodological approach in synthesis of **PET[B]-COOH and PET[C/D]-COOH** precursors: A) 2- ethoxyethanol, KOH, toluene, 120 °C, 4 h; B) DCC/DMAP, CH_2Cl_2, r.t., 12 h; C) H_2 (balloon), Pd/C, 1,4- dioxane, r.t., 6 h.

Experimental procedures

4-((2-ethoxyethoxy)carbonyl)benzoic acid (4): Potassium hydroxide (0.36 g; 6.4 mmol; 1 eq) was dissolved in 2- ethoxyethanol (0.6 mL; 6.4 mmol; 1 eq) with stirring and gentle heating. Then, solution of *bis*(2-ethoxyethyl)terephthalate **1** (Chase ,1963; Djapovic et al., 2021) (2.00 g; 6.4 mmol; 1 eq) and toluene (30.0 mL) was added, and reaction mixture was heated at 120 °C for 4h. After cooling to room temperature, the reaction mixture was filtered, and the remaining filter cake of the ionic salt was washed with EtOAc. After the filter cake was well dried, it was dispersed in water (25.0 mL), and then the aqueous solution was washed with EtOAc (3 x 15.0 mL). The aqueous solution was acidified with HCl (conc.) to pH = 2 and then the acid was extracted from the aqueous layer with EtOAc. The organic layer was dried over anhydrous Na_2SO_4, the solvent was evaporated using a vacuum evaporator. 0.71 g **(47%)** of the acid **PET[B]-COOH** was obtained in the form of a white, crystalline substance (mp 88-90 °C). ^{1}H NMR (400 MHz, $CDCl_3$): δ_H 8.15 – 8.09 (m, 4H), 4.52 (m, 2H), 3.81 (m, 2H), 3.62 (q, J = 7.0 Hz, 2H), 1.25 (t, J = 7.0 Hz, 3H). ^{13}C NMR (101 MHz, $CDCl_3$): δ_C 170.4, 165.9, 134.8, 133.3, 130.2, 129.9, 68.5, 66.9, 64.7, 15.2. IR (ATR) ν_{max}: 3063, 2977, 2863, 2670, 2553, 1712, 1688, 1575, 1510, 1428, 1355, 1276, 1130, 1112, 731. HRMS (ESI): m/z $[M+Na]^+$ calculated for $C_{12}H_{14}O_5$: 261.0733; found: 261.0734.

Benzyl (2-((4-(methoxycarbonyl)benzoyl)oxy)ethyl) terephthalate (5): A solution of dicyclohexylcarbodiimide (DCC) (576.0 mg; 2.79 mmol; 1.1 eq) in dichloromethane (6 mL) was added dropwise to a cold (0 °C) suspension of monobenzyl terephthalate **4** (651 mg; 2.54 mmol; 1 eq), PET monomer **3** (Djapovic et al., 2021) (570 mg; 2.54 mmol; 1.0 eq), 4-dimethylaminopyridine (DMAP) (155 mg; 1.27 mmol; 0.5 eq) and dichloromethane (12 mL). The reaction was carried out at room temperature for 12 hours. The byproduct dicyclohexylurea (DCU) was removed by filtration and precipitate was washed three times with ethyl acetate. The filtrate was concentrated and the residue was purified by dry flash chromatography (SiO_2; eluent: dichloromethane/toluene /ethyl acetate = 60:40:3), to afford 893 mg (**76** %) of benzyl ester **5**, as a white crystals (mp 121-123 °C) (Pénisson and Zahn, 1970). ^{1}H NMR (400 MHz, $CDCl_3$): δ_H 8.16 – 8.07 (m, 8H), 7.46 – 7.34 (m, 5H), 5.38 (s, 2H), 4.70 (s, 4H), 3.94 (s, 3H). ^{13}C NMR (101 MHz, $CDCl_3$): δ_C 166.3, 165.7, 165.7, 135.8, 134.3, 133.7, 133.6, 129.9, 129.8, 129.7, 128.8, 128.6, 128.4, 67.3, 63.14, 63.12, 52.6. IR (ATR) ν_{max}: 2963, 1717, 1581, 1505, 1454, 1437, 1412, 1344, 1275, 1131, 1106, 1022, 975, 728. HRMS (ESI): m/z $[M+Na]^+$ calculated for $C_{26}H_{22}O_8$: 485.1207; found: 485.1206.

4-((2-((4-(methoxycarbonyl)benzoyl)oxy)ethoxy)carbonyl)benzoic acid (6): The catalytic amount of 10% palladium on charcoal was added into the solution of benzyl ester **5** (665.2 mg; 1.44 mmol) in 1,4- dioxane (14 mL). The hydrogenolysis of protected oligomer was performed under hydrogen atmosphere, with rubber balloon filled with hydrogen, at room temperature for 6 h. The reaction mixture was filtered through a plug of celite, solvent was removed in *vacuo*, to afford 492 mg (**92** %) of **PET[C/D]-COOH**, as a white crystals (mp 192-194 °C) (Pénisson and Zahn, 1970). ^{1}H NMR (400 MHz, DMSO-d_6): δ_H 8.07 and 8.04 (two overlapping singlets in ratio 4:4, 8H), 4.66 (s, 4H), 3.87 (s, 3H). ^{13}C NMR (101 MHz, DMSO- d_6): δ_C 166.7, 165.6, 165.2, 165.1, 135.4, 133.8, 133.5, 133.0, 129.7, 129.68, 129.65, 129.5, 63.3, 63.2, 52.7. IR (ATR) ν_{max}: 2964, 2906, 2670, 2544, 1719, 1688, 1579, 1507, 1431, 1410, 1340, 1273, 1130, 1020, 727. HRMS (ESI): m/z $[M+Na]^+$ calculated for $C_{19}H_{16}O_8$: 395.0737; found: 395.0732.

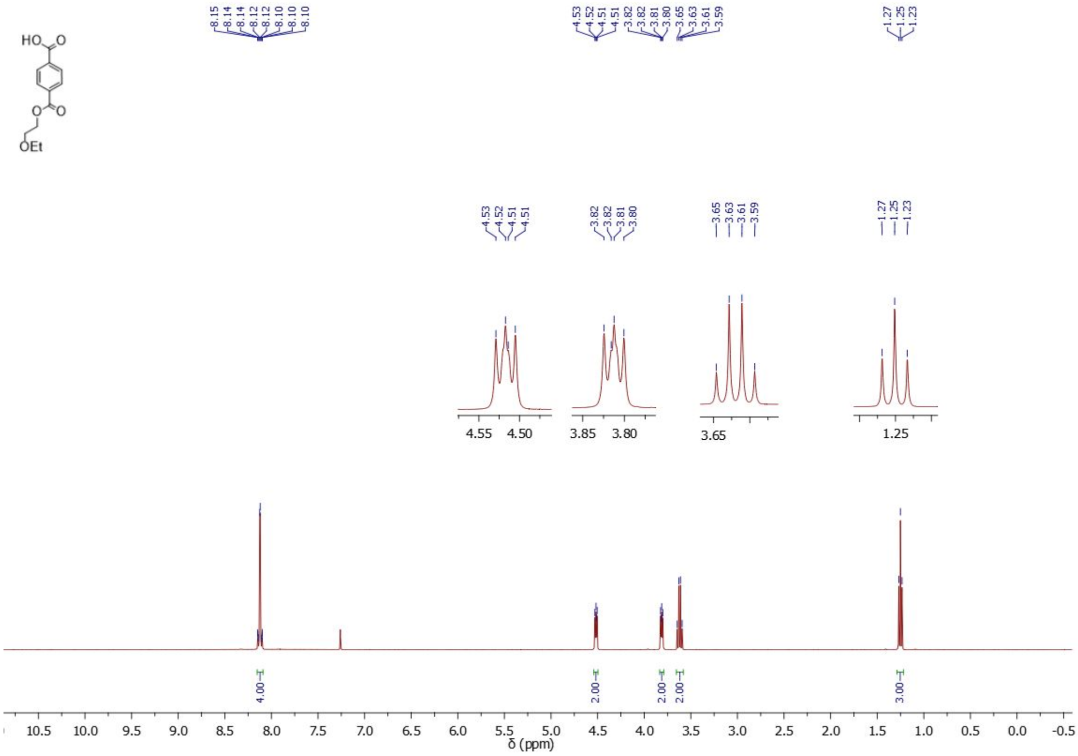

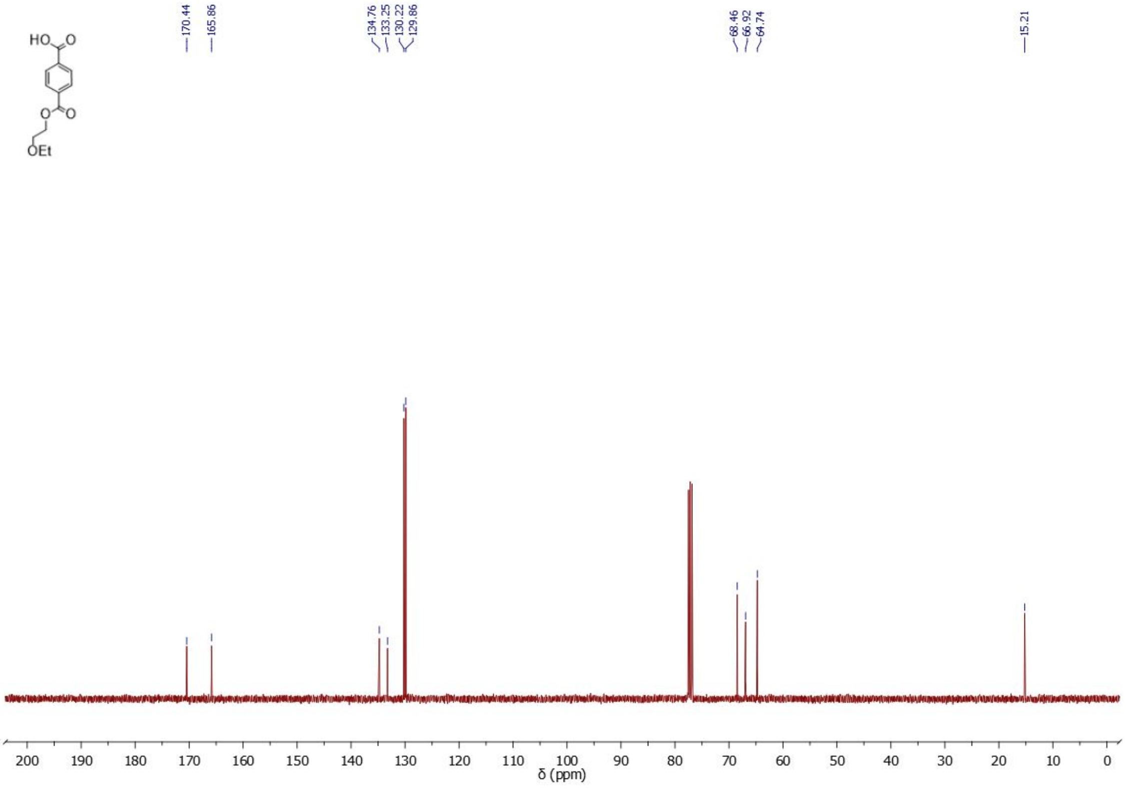

Figure S2. ^{1}H NMR spectrum (400 MHz) of **PET[B]-COOH** recorded in $CDCl_3$ and ^{13}C NMR spectrum (101 MHz) of **PET[B]-COOH** recorded in $CDCl_3$.

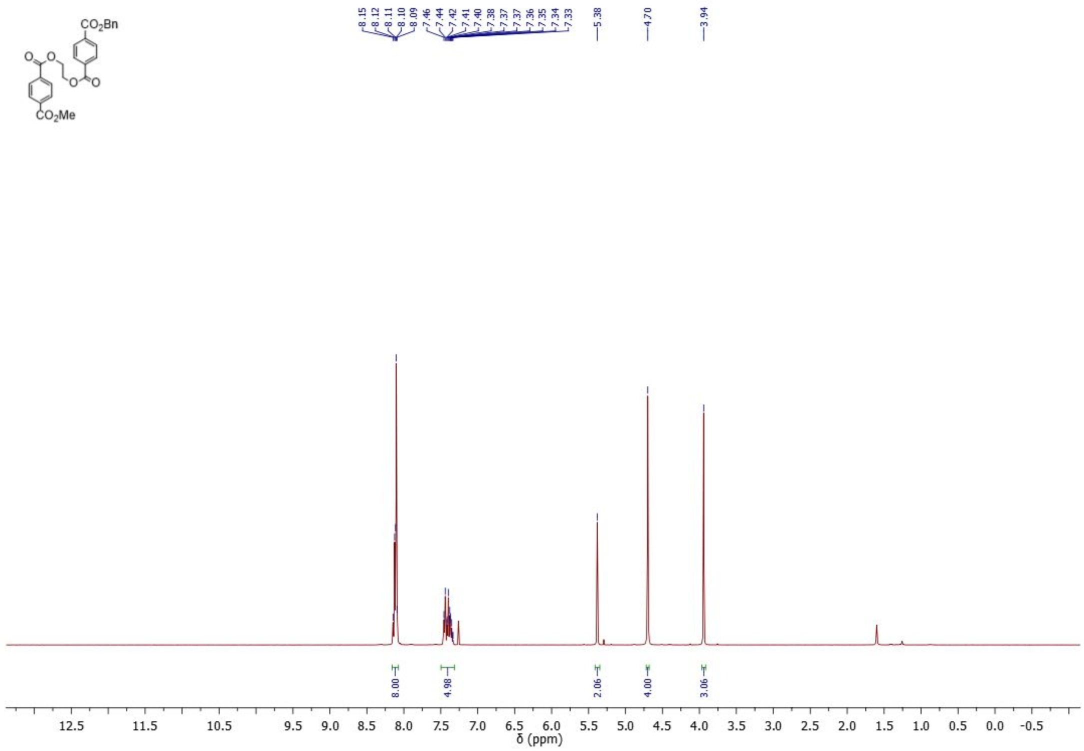

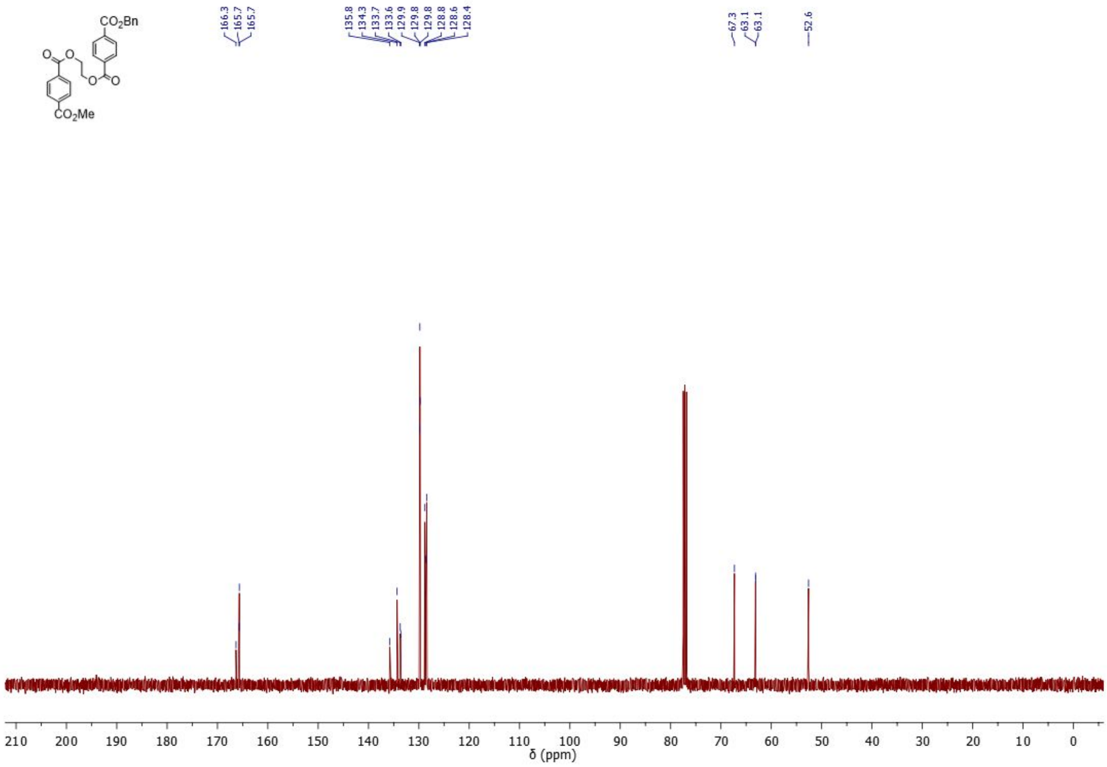

Figure S3. ^{1}H NMR spectrum (400 MHz) of **5** recorded in $CDCl_3$ and ^{13}C NMR spectrum (101 MHz) of **5** recorded in $CDCl_3$.

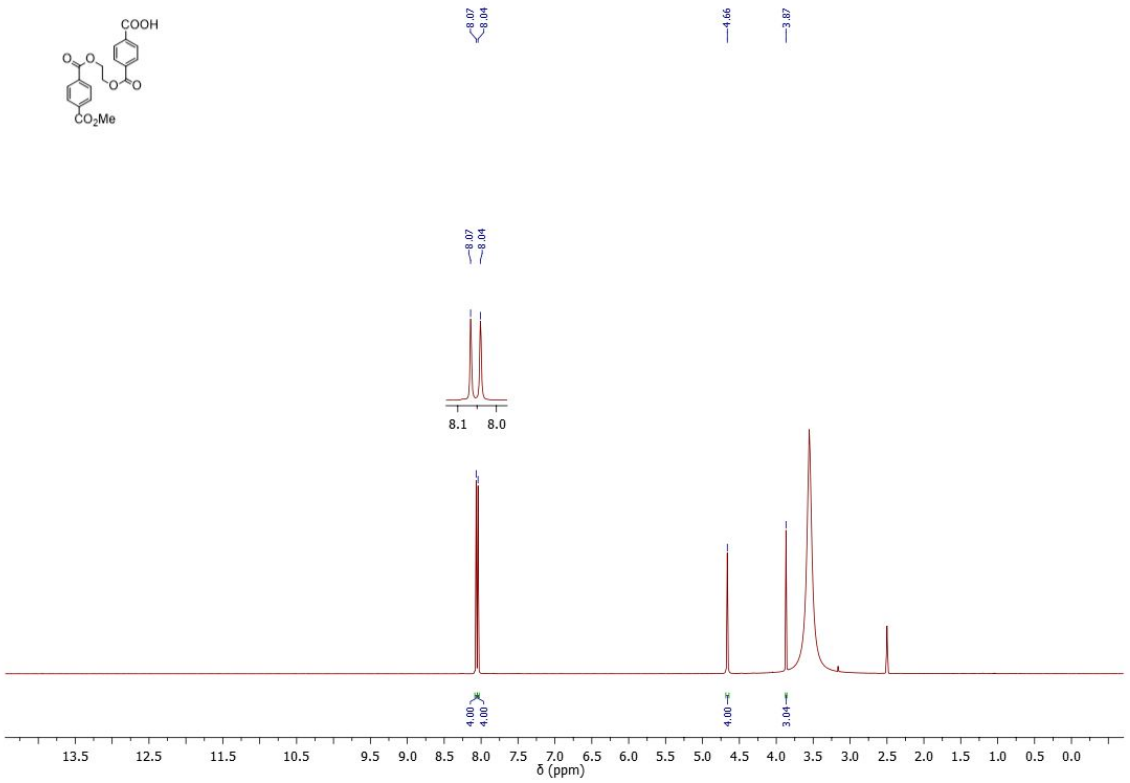

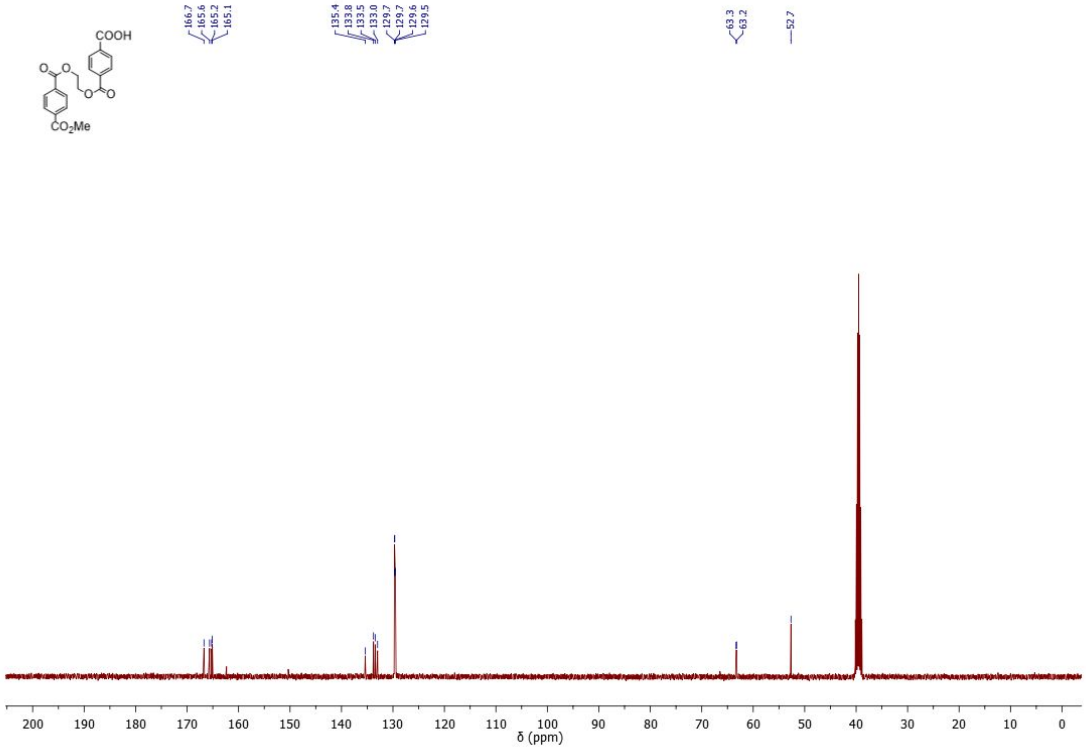

Figure S4. ^{1}H NMR spectrum (400 MHz) of **6** recorded in DMSO-*d6* and ^{13}C NMR spectrum (101 MHz) of **6** recorded in DMSO-*d6*.

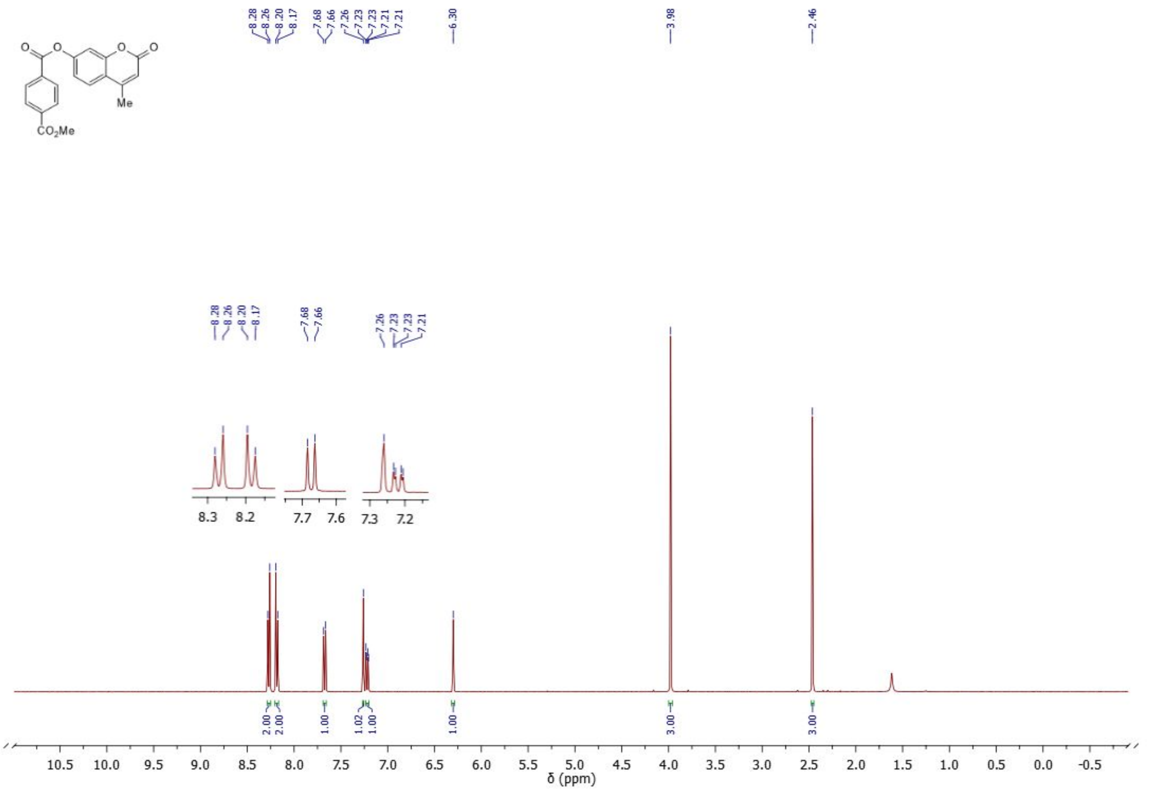

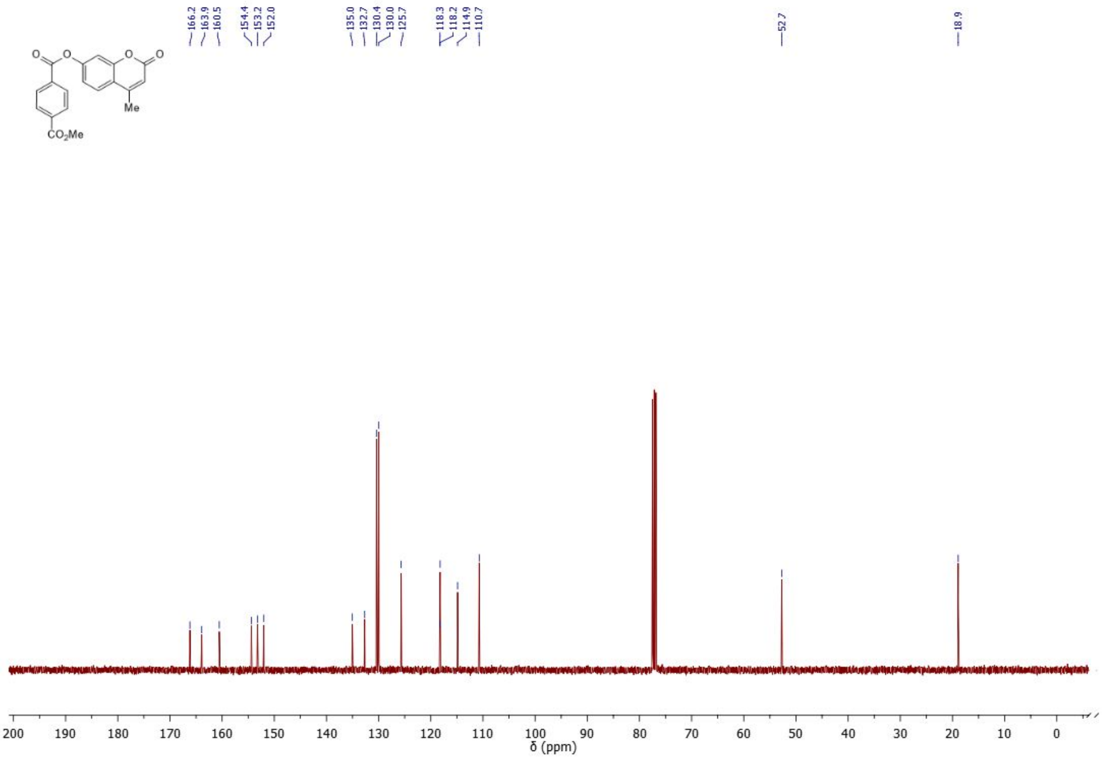

Figure S5. ^{1}H NMR spectrum (400 MHz) of **mUPET1 (A)** recorded in $CDCl_3$ and ^{13}C NMR spectrum (101 MHz) of **mUPET1 (A)** recorded in $CDCl_3$.

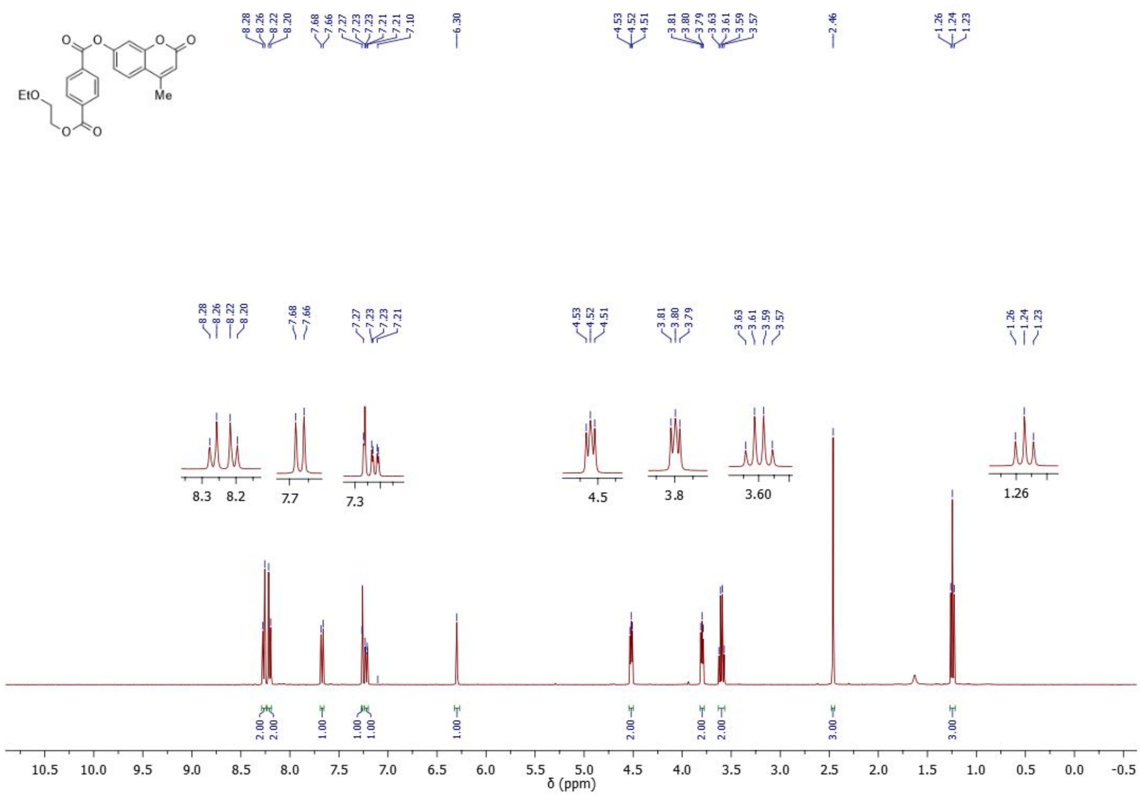

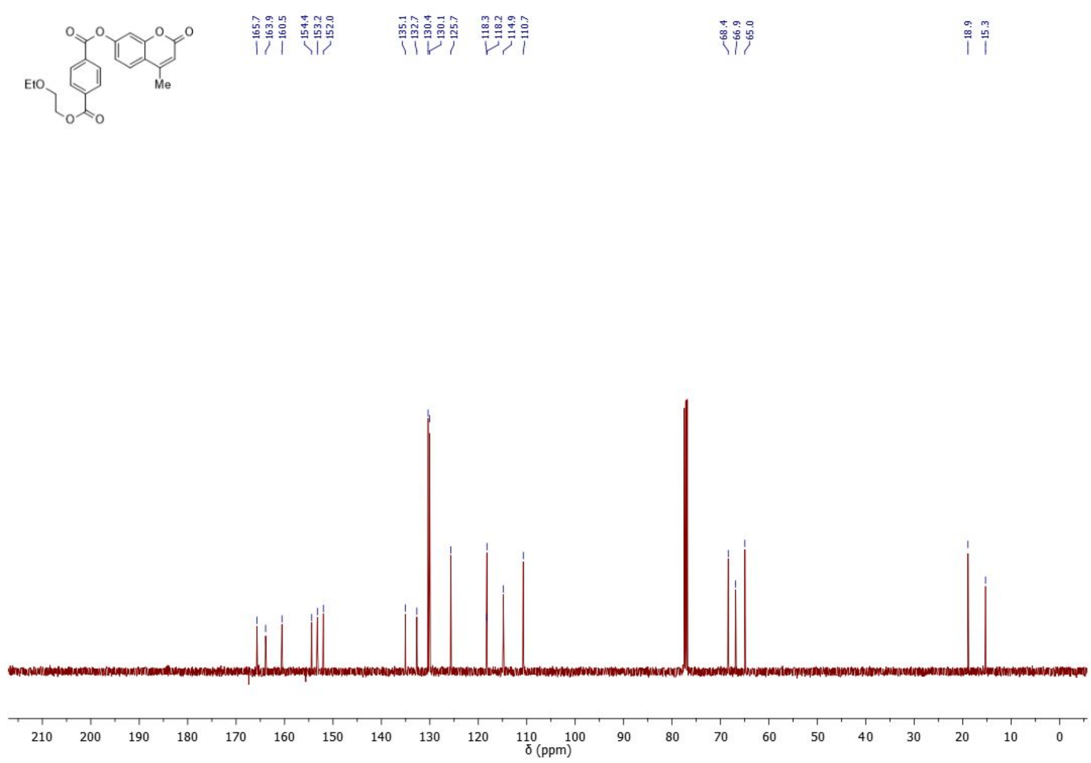

Figure S6. ^{1}H NMR spectrum (400 MHz) of **mUPET2 (B)** recorded in $CDCl_3$ and ^{13}C NMR spectrum (101 MHz) of **mUPET2 (B)** recorded in $CDCl_3$.

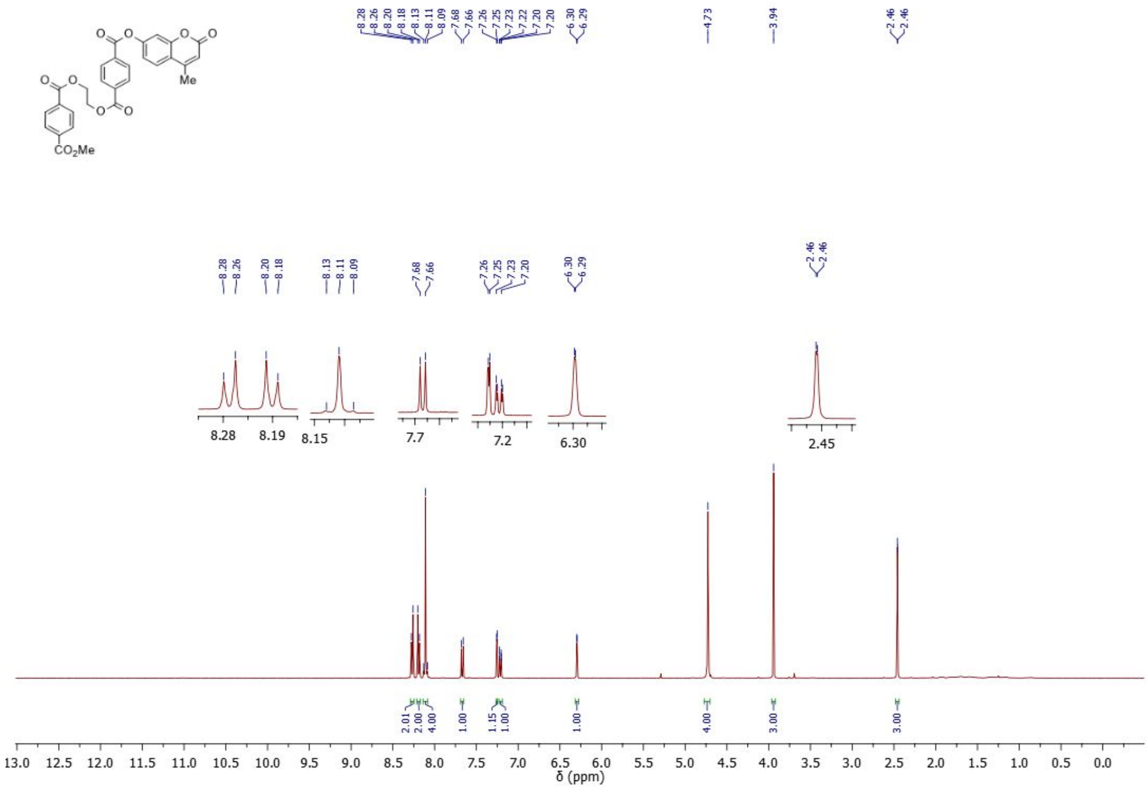

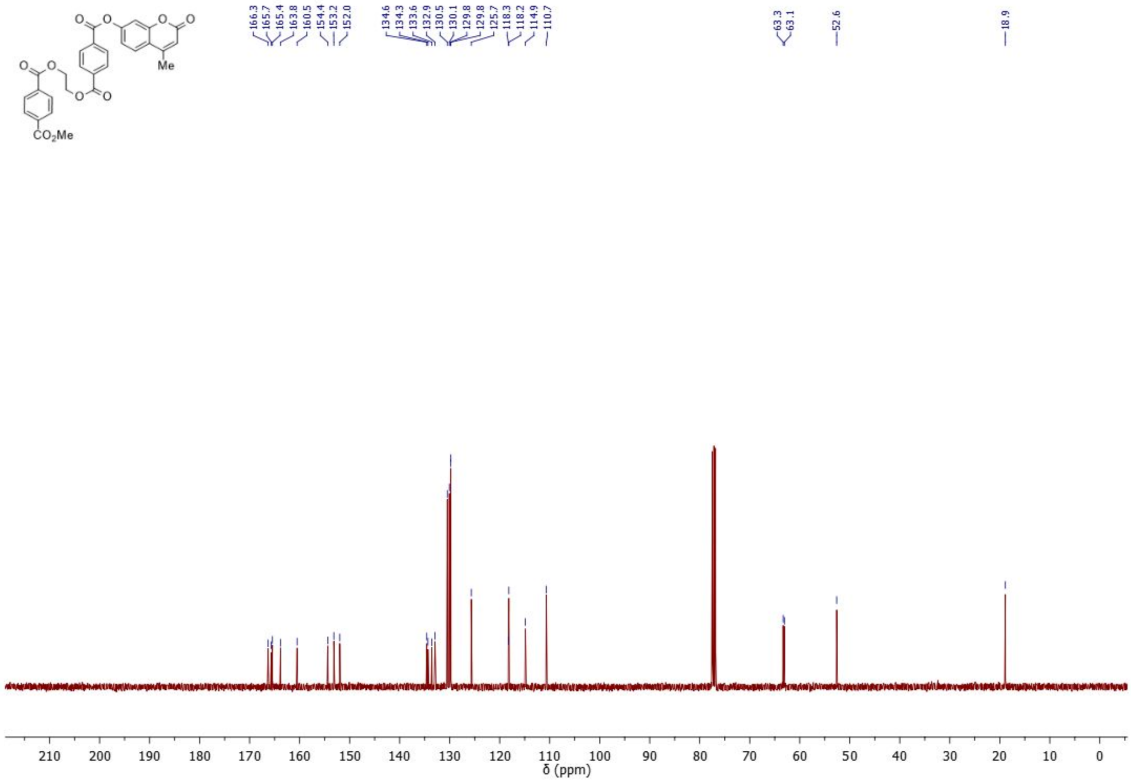

Figure S7. ^{1}H NMR spectrum (400 MHz) of **mUPET3 (C)** recorded in $CDCl_3$ and ^{13}C NMR spectrum (101 MHz) of **mUPET3 (C)** recorded in $CDCl_3$.

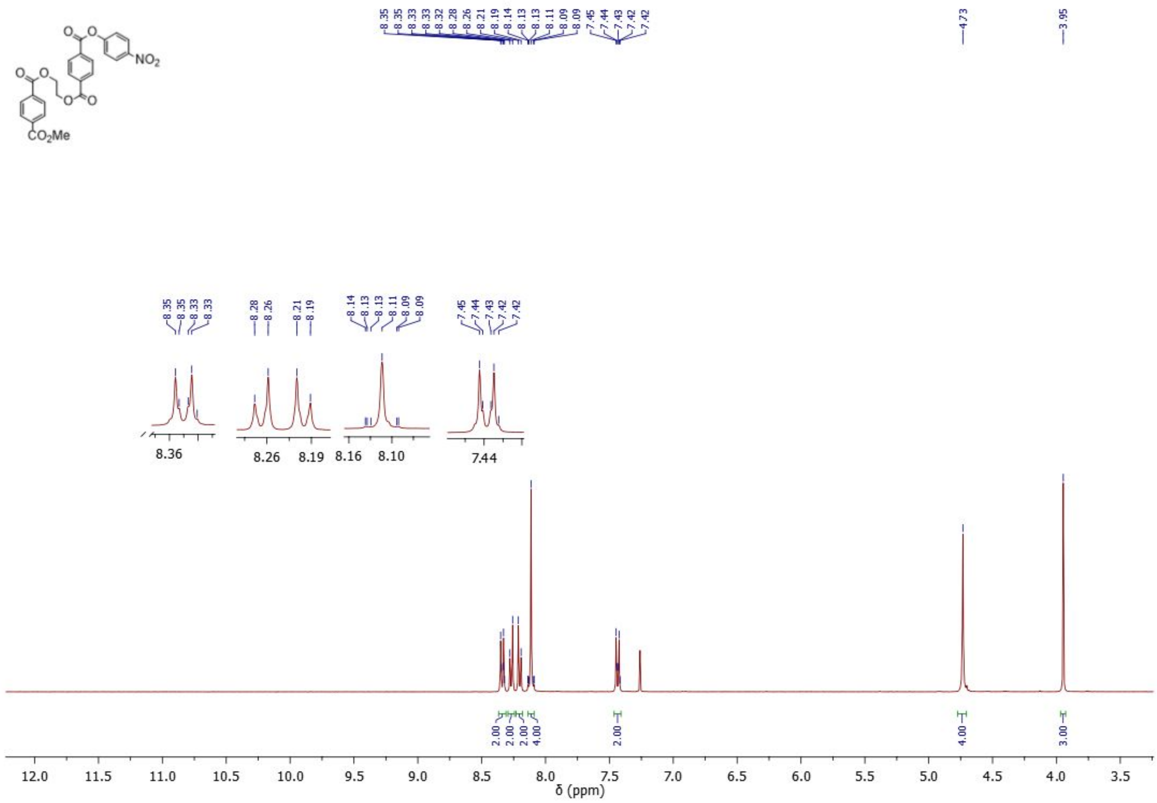

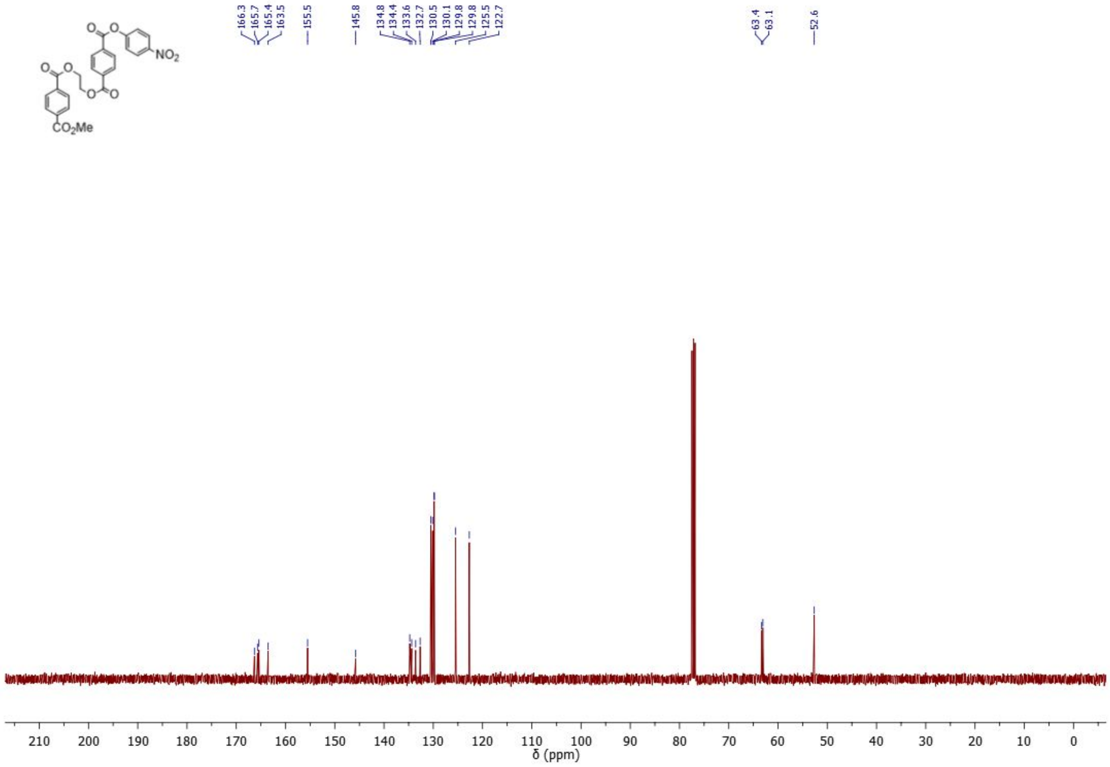

Figure S8. ^{1}H NMR spectrum (400 MHz) of ***p*-NPhPET3 (D)** recorded in $CDCl_3$ and ^{13}C NMR spectrum (101 MHz) of ***p*-NPhPET3 (D)** recorded in $CDCl_3$.

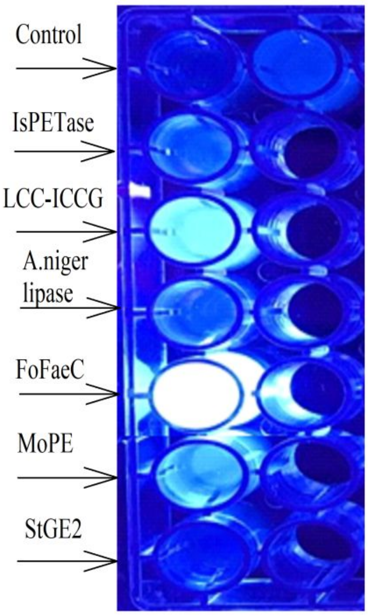

Figure S9. Qualitative assay using 100 µM of mUPET2 as a substrate. The reactions were conducted in a total volume of 100 µL, using 0.1 M phosphate buffer at pH 7. After the addition of 10 µL of each enzyme, the plate was left to incubate for 30 seconds at room temperature before being exposed to UV light.
